# Sources of Variability in the Prospective Relation of Language to Social, Emotional, and Behavior Problem Symptoms: Implications for Developmental Language Disorder

**DOI:** 10.1037/abn0000691

**Published:** 2021-08

**Authors:** Shaun K. Y. Goh, Sarah Griffiths, Courtenay F. Norbury, SCALES Team

**Affiliations:** 1 Centre for Research in Child Development, Office of Education Research, National Institute of Education, Nanyang Technological University; 2 Psychology and Language Sciences, University College London; 3 Department of Special Needs Education, University of Oslo

**Keywords:** language disorder, emotional problems, behavioral problems, peer problems, prospective cohort

## Abstract

Children with developmental language disorder (DLD) are at risk for social, emotional, and behavioral (SEB) maladjustment throughout development, though it is unclear if poor language proficiency per se can account for this risk as associations between language and SEB appear more variable among typical-language children. This study investigated whether the relationship between language and SEB problems is stronger at very low levels of language and considered confounders including socioeconomic status, sex, and nonverbal intelligence. These were examined using a population-based survey design, including children with a wide range of language and cognitive profiles, and assessed using the Strengths and Difficulties Questionnaire and six standardized language measures (*n* = 363, weighted *n* = 6,451). Structural equation models adjusted for prior levels of SEB revealed that the relationship of language at age 5–6 years to SEB at 7–9 years was nonlinear. Language more strongly predicted all clusters of SEB at disordered language levels relative to typical language levels, with standardized betas of −.25 versus .03 for behavioral, −.31 versus −.04 for peer, and .27 versus .03 for prosocial problems. Wald tests between these pairs of betas yielded *p* values from .049 to .014. Sex moderated the nonlinear association between language and emotional symptoms. These findings indicate a clinical need to support language development in order to mitigate against problems of SEB and to carefully monitor the mental health needs of children with DLD, particularly in the context of multiple, and potentially sex-specific, risks.

## General Scientific Summary

Children with a marked deficit in language have higher rates of behavioral and emotional disorders in later life. However, studies among typically developing children do not always find a link between language and symptoms of emotional behavioral disorders. This study supports the notion of unequal risk, where the risk is amplified for children with very low levels of language.

Children with developmental language disorder (DLD) are consistently reported to be at elevated risk for social, emotional, and behavioral (SEB) maladjustment (Curtis et al., 2018; Yew & O’Kearney, 2013). DLD is the consensus term for a condition characterized by deficits in vocabulary, grammar, and/or discourse skills and incorporates the condition previously known as “specific language impairment” (Bishop et al., 2017). DLD affects approximately 7% of the school-aged population (Norbury et al., 2016; Tomblin et al., 1997) and persists into adulthood (Johnson et al., 2010). It is critical to understand the mechanisms underpinning the association between DLD and SEB maladjustment in order to inform intervention approaches.

Meta-analyses of prospective longitudinal (Yew & O’Kearney, 2013) and cross-sectional studies (Curtis et al., 2018) comparing children with DLD to children with typical language have found moderate increases in SEB symptoms in children with DLD. This is the case for both internalizing (anxiety, depression) and externalizing (conduct problems, attention deficits) problems. It has been estimated that between 32.7% and 42.8% of children with DLD meet clinical cutoffs for SEB concern by adolescence (Beitchman et al., 2001; Snowling et al., 2006). However, it is unclear whether it is language deficits per se that are driving this relationship or whether the relationship reflects the presence of other variables that associate with both SEB and DLD, such as lower nonverbal cognitive ability, socioeconomic disadvantage, and male sex. Furthermore, few prospective studies have taken prior levels of SEB into account, limiting conclusions about the direction of the relationship between DLD and SEB maladjustment. If language alone were driving the relationship between DLD and SEB, we would expect a prospective relationship between language measures and SEB symptoms across the spectrum of language ability. Studies of typically developing children provide evidence of an association, but the effect size is typically modest (Chow & Wehby, 2018). For example, Petersen et al. (2013) reported a small but significant (*B* = .01 to .02) prediction from language to behavior, which maintained after inclusion of covariates such as socioeconomic status (SES), academic achievement, sex, and prior levels of behavioral and inattention-hyperactivity problems. The developmental relationship was in one direction only—language predicted behavior, but behavior was not longitudinally predictive of later language, consistent with the hypothesis that language deficits have a causal role in SEB maladjustment. However, the small effect size raises questions about the mechanistic role of language in developing SEB competence (Curtis et al., 2018; Yew & O’Kearney, 2013).

One possible explanation for the smaller effect sizes observed in population cohorts, relative to studies comparing children with and without DLD, is that the relationship between language and SEB may be nonlinear. Stronger relationships at the tail of the language distribution may indicate that limited language competence prevents the development of skills that promote SEB adjustment—for example, using language to regulate emotion or develop supportive peer relationships. However, once a sufficient level of language is reached to allow development of such skills, greater language competence may not provide additional SEB benefit. Comparison of correlations at different points on the language continuum supports this view. For example, Plomin et al. (2002) reported that correlations between language and SEB in preschoolers (aged 2–4 years) were larger for children scoring below the 10th percentile on language, relative to the rest of the population (total sample *r* = .05 to .18, 10th percentile = .09 to .32). Similarly, Conway et al. (2017) found that the relationship between language and behavior in 2–4-year-olds was strongest at below-average levels of language proficiency and not evident at average or above-average levels. These findings suggest that a “threshold” account, in which the risk for poor outcome is greatest at extreme levels of language deficit, may better characterize language risk for adverse SEB.

Support for a threshold account is currently inconclusive and limited by focus on underfives; we lack data on the relationship between language and SEB in older children with more persistent language disorder. A second limitation concerns sampling; larger population studies tend to focus on cohorts with average or above-average language skills, whereas studies of children with DLD focus predominantly on clinical referrals that are subject to Berkson’s (1946) bias, where children with co-occurring conditions are more likely to be referred for clinical services, even if the two conditions are unrelated in the population. In addition, few studies have taken prior levels of SEB into account, which may inflate estimates of a language-behavior relationship. Hence, whether a threshold account would be supported in a population-derived sample of older children with DLD remains an empirical question.

Alternatively, the stronger association between language and SEB in clinical samples could reflect the presence of other variables that associate with both SEB and DLD, such as lower nonverbal cognitive ability, socioeconomic disadvantage, and male sex. Nonverbal cognitive skills are thought to function as a general protective factor for mental health, through either reduced exposure to or the buffering of negative life stressors (Caspi et al., 2014). Notably, all meta-analyses and many primary studies have used diagnostic criteria for “specific language impairment” that exclude children with comorbidities or lower nonverbal ability scores. Consequently, there is little data regarding the potential moderating effect of nonverbal IQ (NVIQ) on language-SEB relationships, and existing evidence is inconsistent. In one longitudinal study of 12,099 5-year-old children, only those with co-occurring language *and* cognitive deficits were at elevated risk of poor mental health in adulthood; language deficit in the context of adequate nonverbal cognitive ability did not elevate risk (Law et al., 2009; see also Snowling et al., 2006). In contrast, results from a large epidemiological sample found that children with language impairment experienced similar levels of SEB outcome, regardless of cognitive ability (Tomblin & Nippold, 2014). Hence, the role of nonverbal cognitive ability in SEB outcome remains unclear.

Socioeconomic disadvantage is consistently associated with both language (Noble et al., 2007) and SEB outcomes (Kalff et al., 2001). Thus, their co-occurrence may be particularly potent for SEB outcomes in children with DLD. For example, SES amplified the association between vocabulary and SEB in a community sample (Yew & O’Kearney, 2015a, 2015b) and warrants further investigation as a moderator.

Numerous studies have highlighted sex differences in DLD and SEB problems, with boys overrepresented in clinical samples of DLD, and externalizing and attention-deficit/hyperactivity disorder types of SEB (Ford et al., 2003; Mouridsen & Hauschild, 2010; Polanczyk et al., 2015; Whitehouse, 2010). Far less is known about how sex moderates the relationship between language and SEB. For instance, girls with DLD have been found to be at elevated risk of emotional symptoms in middle childhood (Beitchman et al., 2001), and adolescent boys with DLD have reported greater symptoms of depression (Conti-Ramsden & Botting, 2008), while in studies with larger samples, no sex differences have been evident (Yew & O’Kearney, 2015a, 2015b). Taken together, these studies suggest the possibility of symptom moderation by sex in later childhood, though no previous study has investigated whether sex moderates the longitudinal relationship between early language and later SEB.

The multitude of methods used to measure language and SEB makes it difficult to compare studies, especially when there is an attempt to relate specific aspects of language (receptive or expressive) to different types of SEB outcome (internalizing vs. externalizing problems). Two meta-analyses (Chow et al., 2018; Chow & Wehby, 2018) reported generally stronger associations between receptive language and any SEB outcome, relative to associations with expressive language, though individual studies have varied considerably in the strength and specificity of subtype analyses. In the current study, we employed latent variables, which permit comparison of underlying constructs over time, undiluted by the measurement error associated with specific assessments, yielding more precise estimates (Putnick & Bornstein, 2016).

The current study investigates the prospective association of language to SEB utilizing an intensively profiled cohort of children with and without language disorder. To guard against Berkson’s bias, all children were recruited from community schools using a population-based survey design. This longitudinal sample represents children starting mainstream education in Surrey in Southeast England in reception year (ages 4–5) and includes a wide range of language and cognitive profiles. Children were identified as having language disorder on the basis of standardized tests of language and functional impact on education performance (e.g., Norbury et al., 2016); however, the study was designed to examine the role of language in SEB development across the full spectrum of ability, and therefore our analyses employed language as a continuous variable.

To our knowledge, this is the first study to empirically and systematically evaluate three sources of variability influencing prospective associations between language and SEB. First, we considered language modality by testing a measurement model that included six standardized measures of language. Second, we examined the threshold account by modeling the prospective association of language at Year 1 (age 5;1 to 6;10) to SEB problem outcomes at Year 3 (age 7;1 to 9;3) at different levels of language, adjusting for prior SEB symptoms. Finally, we considered potential moderators of this association, including nonverbal cognition, socioeconomic status, and male sex.

## Method

### Participants

**Figure 1 fig1:**
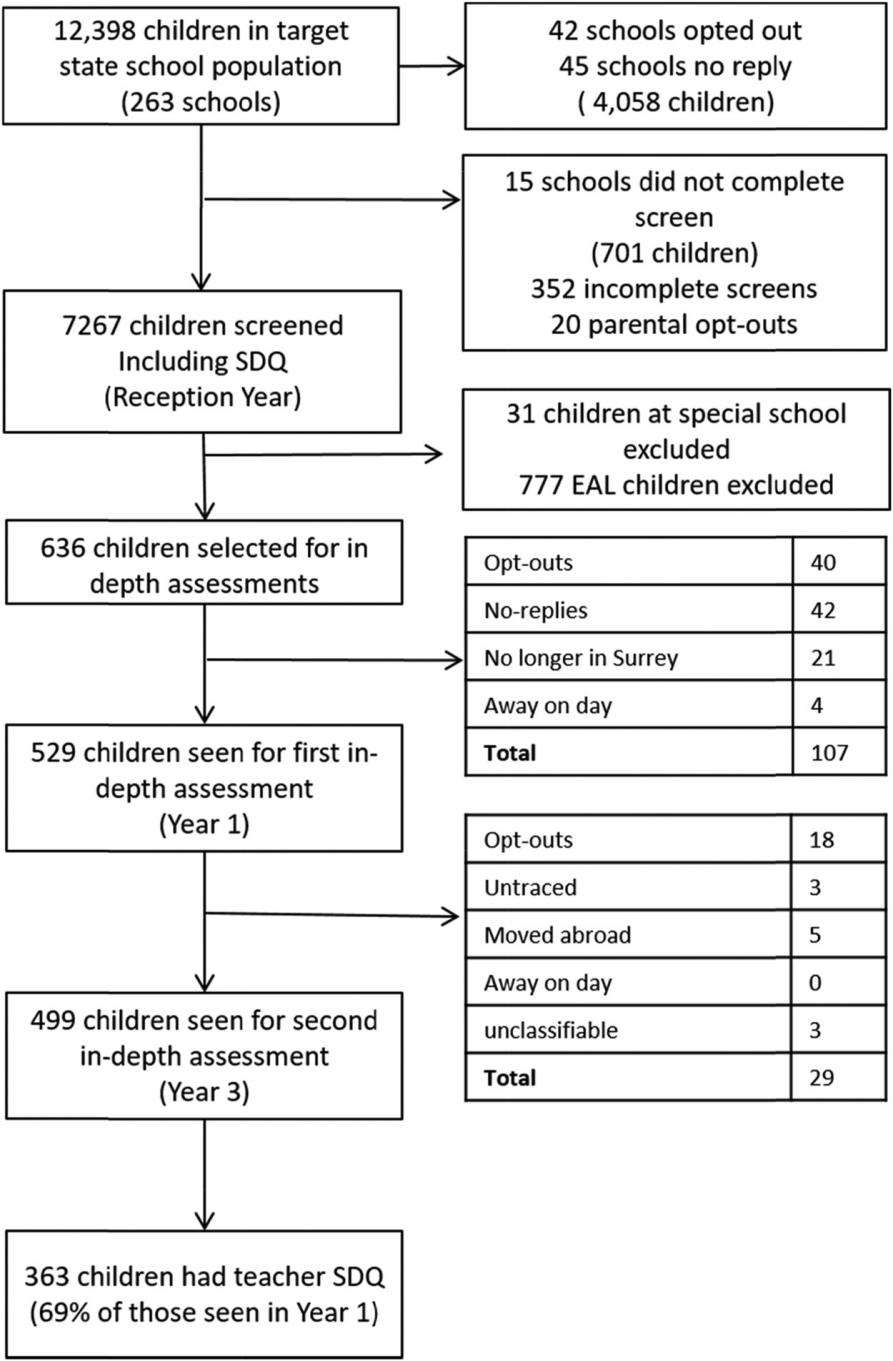
Recruitment Flow Chart of the Surrey Communication and Language in Education (SCALES) Study *Note*. One of the 529 children seen for the first in-depth assessment did not provide sufficient data to be included in the analysis. Therefore, data is available for 528 children. EAL = English as an additional language; SDQ = Strengths Difficulties Questionnaire.

Participants were drawn from the Surrey Communication and Language in Education Study (SCALES; Figure 1), a longitudinal cohort study of language development and disorder (see Norbury et al., 2016, for details). Data from reception, Year 1, and Year 3 were included in the current study. In the first phase, teacher-rated language and SEB questionnaires were available for 7,267 children (59% of children enrolled in state-funded reception classes in Surrey during 2011/2012) aged between 4;9 and 5;10 and predominately of White ethnicity (*n* = 5,979, 82.3%). Socioeconomic status was estimated using the Income Deprivation Affecting Children Index (IDACI) rank score, ranging from 1 for the most deprived area in the United Kingdom to 32,482 for the least deprived, and in SCALES, the mean rank was 21,364.35 (*SD* = 7,755.31), relative to the national average of 16,241. There were no differences between participating schools and remaining schools in terms of neighborhood deprivation, the numbers of children on role with special educational needs, or provision of free school meals (Norbury et al., 2016).[Fig-anchor fig1]

Monolingual children were selected for in-depth assessment (Year 1) using stratified random sampling accounting for language proficiency at screen, sex, and season of birth. We invited all children reported to have “no phrase speech” (defined as producing only single words or two combinations, *n* = 48), 40% of children with teacher-rated low language proficiency (scores < 14th percentile for season of birth on teacher-rated Children’s Communication Checklist—Short), and 4% of all remaining children. We oversampled girls to ensure equal numbers of both sexes. Our weighting procedures took account of this sampling design (i.e., boy scores carry greater “weight”), and therefore weighted estimates reflect the screened population (see online Supplemental Materials S1 for weighted histograms).

Of the 636 children invited, 529 monolingual children (83% of those invited) were profiled using multiple assessments of language, behavior, and cognition (50.2% male; Norbury et al., 2016). Children were aged between 5;1 and 6;10, were predominantly of White ethnicity (*n* = 476; 90.2%; see online Supplemental Materials S1 for breakdown), and had a mean rank IDACI score of 21,366.39 (*SD* = 7,763.54). Assessments were conducted at the child’s school and lasted approximately 2 hr (with breaks). Children met the criteria for language disorder if they scored −1.5 standard deviations or below on two out of five language composites (vocabulary, grammar, narrative, receptive language, and expressive language; Norbury et al., 2016). One hundred thirty-six children (25.8% total sample) met research criteria for language disorder in Year 1 (age 5–6 years). Of these, 45 had an existing known diagnosis associated with language disorder, while the remaining 91 children were classified as having DLD (Bishop et al., 2017). Fourteen children had an autism diagnosis, and 15 children had a rare biomedical condition reported by parents or teachers (e.g., Down’s syndrome, neurofibromatosis). An additional 16 children were identified as having intellectual disability based on receiving scores of less than −2 standard deviations below the mean on standardized NVIQ tests. All other children were deemed to have typical language development (*n* = 392).

In Year 3, 95% (*n* = 499) of the cohort were reassessed (Norbury et al., 2017), with teacher reports of SEB symptoms using the Strengths and Difficulties Questionnaire (SDQ; Goodman, 1997) available for 363/499 children (73% of reassessed cohort, 68.6% of children profiled at Year 1). They were aged between 7;1 and 9;3, were predominantly of White ethnicity (*n* = 324, 89.3%), and had a mean IDACI rank score of 21,372 (*SD* = 7783.12).

### Sampling Weights and Missing Data

Inverse probability weighting is often utilized in large longitudinal studies to yield population representative samples by calculating weights that account for selective participation and adjusting them for inevitable nonresponse in subsequent waves. Likewise, inverse probability weighting is utilized in SCALES, as detailed elsewhere (Gooch et al., 2019; Vamvakas et al., 2019). In brief, weights were constructed as the inverse of the probability of inclusion in the study from a logistic regression model fit to the entire screened population of 6,459 monolingual children attending mainstream schools; this model estimated the probability of inclusion for in-depth assessment at Year 1, with predictor variables including sex, season of birth, and scores on the Children’s Communication Checklist—Short. These weights were further adjusted for differential nonresponse/missing data at Year 3 by estimating a second logistic regression model fit to 529 children selected for in-depth assessment at Year 1. This model utilized predictors of missingness on the SDQ data (*n* = 125) including IDACI rank score, SDQ total difficulties score, pupils on school role, percentage of children in school with special educational needs, and percentage receiving free school meals. The final sampling weights were the multiplication of the inverse of the probabilities from both logistic models (Vamvakas et al., 2019). There were no differences between children with (*n* = 363) and without (*n* = 125) teacher-rated SDQ on age, sex, nonverbal cognition, DLD status, SDQ scores at reception, or SES (IDACI rank scores; online Supplemental Materials S2). Hence, the weighted models are representative of the monolingual cohort from which this sample was drawn.

### Consent Procedures

Consent procedures and study protocol were developed in consultation with Surrey County Council and approved by the Research Ethics Committee at Royal Holloway, University of London, where this study, The Surrey Communication and Language in Education Study, originated. Ethical approval for continued data storage and analysis is provided by the University College London Research Ethics Committee (9733/002). For the screening phase, opt-out consent was employed as data could be provided anonymously; 20 families opted out. In the second phase, written, informed consent for two episodes of direct assessment, including teacher report of child language and behavior, was obtained from parents or legal guardians of participants. Prior to assessment in Year 3, families received an additional information sheet and the option to withdraw from the study; 18 families withdrew, five moved abroad, three could not be contacted, and three provided insufficient data at test for diagnostic classification. Of the 29 children (19 male) not included in follow-up, 22 had been classified as “typically developing” in Year 1 and had no evidence of language, learning, or behavioral difficulties.

### In-Depth Assessment

#### Social, Emotional, and Behavioral Problems

Teachers completed the SDQ (Goodman, 1997) at reception and in Year 1. The SDQ is a well-validated questionnaire rating 25 items tapping SEB strengths and weakness across five subscales (peer problems, emotional symptoms, conduct problems, inattention/hyperactivity, and prosocial), each rated on a 3-point scale (e.g., 0 = *not true*, 1 = *somewhat true*, 2 = *certainly true*, possible score range 0–10). Internal consistency for teacher ratings is variable, with Cronbach’s alpha for this sample ranging from .67 (peer problems) to .87 (hyperactivity). This is consistent with pooled reliability estimates (Stone et al., 2015), .63 (peer problems) to .83 (hyperactivity), and pooled test–retest reliability, .72 (emotional problems) to .85 (hyperactivity). SDQ scores at reception (age 4–5) and Year 3 (ages 7–9) were utilized in this study.

#### Receptive/Expressive One-Word Picture Vocabulary Tests (R/EOWPVT-4; Martin & Brownell, 2011)

ROWPVT and EOWPVT require word-to-picture matching and picture naming tests, respectively, with possible scores ranging from 0–190. Test–retest reliability is .97 for both measures, and internal consistency for ages 5 to 8 years is excellent (Cronbach’s alpha = .94–.97).

#### Test of Reception of Grammar—Short Form (Bishop, 2003)

Forty of the original 80 test items were included in which children heard a sentence such as “The ball that is red is on the pencil” and were asked to select the corresponding picture out of a choice of four. If a child answered incorrectly on six consecutive items, then the test was discontinued. Scores for this task range from 0 to 40, with excellent agreement between short and long forms in pilot testing, *r*(17) = .88.

#### School-Age Sentence Repetition Imitation Test—English (SASIT-E32)

The SASIT-E32 (Marinis et al., 2011) asks the child to repeat prerecorded sentences of increasing length and grammatical complexity, played over headphones (possible score 0–32). Interrater reliability for scoring is excellent, .98 (Chiat & Roy, 2013).

#### Assessment of Comprehension and Expression 6–11 (Adams et al., 2011)

In narrative recall, the child was asked to listen to a prerecorded story with accompanying pictures displayed on a laptop computer. After listening to the story, the child was asked to tell the story in their own words with the pictures displayed. The child was awarded 1 point for a maximum of 35 propositions accurately retold. Internal consistency is adequate (Cronbach’s alpha = .73) for children aged 6 to 11 years.

A bespoke measure of narrative comprehension was constructed in which the child was asked to answer 12 (six literal and six inference) questions about the story. Answers were scored on a 3-point scale (0 for an incorrect/no response, 1 for a partially correct response, and 2 points for a complete and accurate response) with a total possible score of 24. All scoring was done by consensus to ensure rater consistency. For all aforementioned language measures, test scores at Year 1 (ages 5–6) were utilized in this study.

#### Nonverbal Ability

Nonverbal ability (NVIQ) was measured using block design and matrix reasoning subtests. These were from the Wechsler Preschool and Primary Scales of Intelligence (3rd U.K. ed.; Wechsler, 2003) in Year 1 (ages 5–6) and the Wechsler Intelligence Scales for Children (4th U.K. ed.; Wechsler, 2004) in Year 3 (ages 7–9).

#### Socioeconomic Status

The IDACI scores were derived from household postcodes and provide an estimate of socioeconomic deprivation (McLennan et al., 2011). Deprivation is defined as households receiving income support, jobseeker’s allowance, working or disabled person’s tax credits, or national asylum support whose equalized income is 60% below national median before housing costs.

### Analytic Strategy

Three sets of latent variable models, examining (a) language, (b) SEB, and (c) language and SEB, were run in Mplus Version 7.4 with the WEIGHT command in order to incorporate inverse probability weights, yielding weighted estimates (model *n* = 363, weighted *n* = 6,451). First, to address measurement issues, we conducted confirmatory factor analysis of scores on the six language measures using the maximum likelihood robust estimator. We asked whether receptive and expressive language factors could be modeled separately to ascertain modality-specific relationships with SEB outcomes (cf. Snowling et al., 2006). In fact, the initial two-factor model was inadmissible due to correlations between the two factors exceeding 1. Instead, a single language factor provided the best fit to the data (χ^2^ = 7.37, *df* = 8, *p* = .497, comparative fit index [CFI] = 1.00, Tucker-Lewis index [TLI] = 1.00, root mean square error of approximation [RMSEA] = .00, 90% CI [.00, .058]), with factor loadings ranging from .60 to .81 across all six measures. The one-factor model had a high reliability of omega of .90, while its factor scores also had a high reliability of .90 (online Supplemental Materials S3), hence minimizing the possibility of attenuation of associations due to measurement error (Rdz-Navarro, 2019).

Second, longitudinal SEB models were then tested for measurement invariance, using the weighted least squares mean and variance (WLSMV) adjusted estimator to account for the ordered-categorical outcomes of the SDQ (Liu et al., 2017). Robust testing of measurement invariance ensures that the underlying construct of SEB is being measured or interpreted in the same way by different respondents at different testing points (Putnick & Bornstein, 2016). The level of strong measurement invariance with thresholds and factor loadings of like items constrained to equality is recommended for unbiased path regression results (Guenole & Brown, 2014). Measurement invariance was sufficient for four of the five subscales of interest (online Supplemental Materials S4). It was inadequate for the hyperactivity subscale, which could not be examined for configural invariance as this model failed to converge. Mean scores on this subscale are reported for information, but we did not model longitudinal relationships between language and hyperactivity as parameter estimates are invalid due to failure to converge, and neither fit indices nor modification indices are available for use to guide post hoc modifications.

Third, language, sex, socioeconomic status, and NVIQ were successively added as predictors of the longitudinal SEB factor models. Language was first entered as the sole predictor to examine its prospective association with SEB after accounting for earlier SEB scores. Next, a nonlinear term (Language Level × Language Level) was added to examine the threshold account. Finally, sex, SES, and NVIQ were included as potential moderators. As interactions with latent variables cannot be specified in the WLSMV estimator (Muthén & Muthén, 2012), the observed variable factor scores from the latent model of language were used instead. All lower-order predictors were mean centered to allow interpretation as main effects. To clarify the nature of significant interactions, they were decomposed through plots of their simple slopes at levels of −1.5 standard deviations and 0 standard deviations. Models that were uniformly above cutoffs for good-fitting models (Hu & Bentler, 1999) of TLI > .95, CFI > .95, and RMSEA < .06 were considered as “close” fit. As it is also important to consider the chi-square test of model fit, models that passed this test in addition to the aforementioned cutoff were considered to be of “good” fit. Models that passed two out of three fit criteria, with the offending index not below less stringent cutoffs of TLI > .90, CFI > .90, and RMSEA < .08 (Bentler & Bonett, 1980; Browne & Cudeck, 1992), were considered as “acceptable” fit.

## Results

Table 1 presents descriptive statistics weighted from the longitudinal cohort of 529 children at Year 1 and of 363 children subsequently reassessed at Year 3. In Year 1, children with DLD obtained lower scores on a nonverbal ability composite, had a higher proportion of boys, and experienced higher levels of socioeconomic deprivation relative to peers with typical language development. DLD children also had persistently elevated symptoms on hyperactivity-inattention, peer, and prosocial subscales, with approximately 50% of DLD children scoring at or above the borderline-abnormal range at Year 3. Aforementioned findings also appear applicable to children with language disorder associated with a known diagnosis (autism, intellectual disability, or other biomedical condition) but were not tested for statistical significance in keeping with the DLD focus of this study. According to the SDQ manual (Youthinmind, 2016), 20% of children aged 4–17 are expected to have a borderline to abnormal range SEB concerns. In SCALES, the percentages of DLD and typical language children scoring in the borderline range appear lower than 20% across SDQ subscales of emotional and conduct problems at Years 1 and 3 (see Table 1).[Table-anchor tbl1]

### Longitudinal Relationships Between Language and SEB

For SDQ subscales in which measurement invariance was established, two-wave lagged path models were constructed (SEB at intake to SEB in Year 3). Year-1 language significantly improved prediction of Year-3 emotional problems but not conduct or peer problems or prosocial behavior, emotional problems β = −.17 (.07), *p* = .009; conduct problems β = .00 (.06), *p* = .941; peer problems β = −.06 (.06), *p* = .332; prosocial β = .05 (.06), *p* = .402, where β refers the standardized beta followed by its standard errors in parentheses. Path diagrams for these models are provided in online Supplemental Materials S6.

**Figure 2 fig2:**
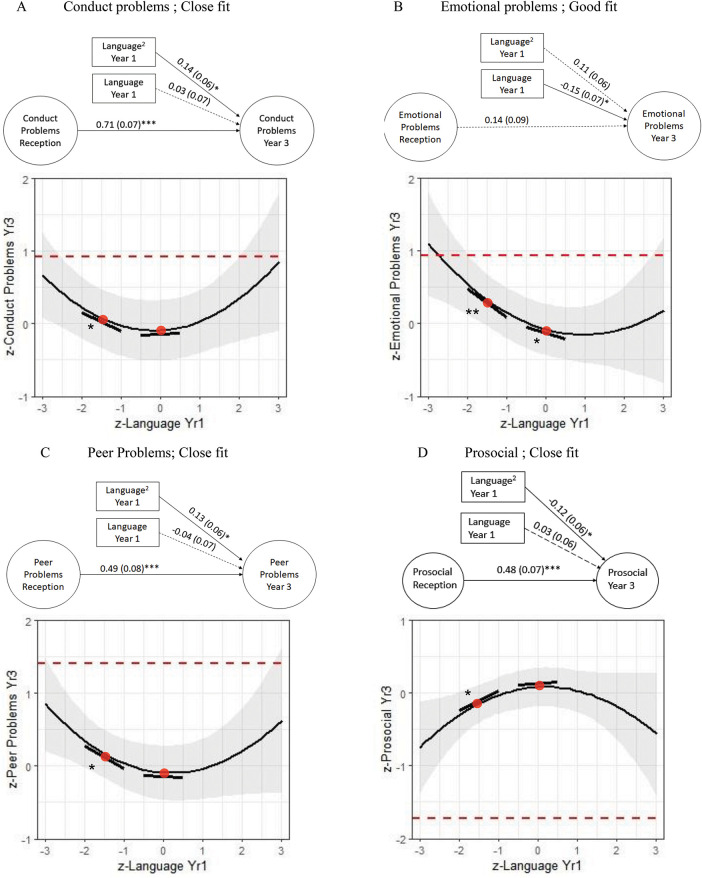
Structural Equation Diagrams of the Contribution of Language to (A) Conduct, (B) Emotional, (C) Peer, and (D) Prosocial Problems Adjusted for Prior Levels With Accompanying Decompositions *Note*. Dotted lines denote borderline highest 20% cutoffs. Models *n* = 363, weighted *n* = 6.451. Fit indices of models are (A) χ^2^ = 94.59 (52), *p* = .000, CFI = .97, TLI = .96, RMSEA = .05, 90% CI [.03, .06]; (B) χ^2^ = 66.18 (53), *p* = .106, CFI = .99, TLI = .99, RMSEA = .03, [.00, .04]; (C) χ^2^ = 93.02 (44), *p* = .000, CFI = .96, TLI = .95, RMSEA = .06, [.04, .07]; (D) χ^2^ = 94.95 (52), *p* = .00 CFI = .99, TLI = .98, RMSEA = .05, [.03, .06]. * *p* < .05. ** *p* < .01. *** *p* < .001. See the online article for the color version of this figure.

A nonlinear quadratic term of language proficiency was then added as a predictor (see Figure 2). This quadratic term predicted Year-3 conduct, peer, and prosocial problems, conduct problems: Language^2^ β = .14 (.06), *p* = .018; peer problems: Language^2^ β = .13 (.06), *p* = .023; prosocial problems: Language^2^ β = −.12 (.06), *p* = .040. The quadratic term also showed a marginal relationship with emotional problems, Language^2^ β = .11 (.06), *p* = .07.[Fig-anchor fig2]

This nonlinear relationship was decomposed in Figure 2; in line with our diagnostic cutoffs, we compared the slope at language scores of -1.5 standard deviations with the slope at the normative language mean of 0 standard deviations. Slopes differed for conduct problems (Wald test = 6.06, 1, *p* = .014), peer problems (Wald test = 4.48, 1, *p* = .034), and prosocial (Wald test = 3.87, 1, *p* = .049).

Slopes at −1.5 standard deviations were of moderate effect size, conduct problems β = −.25 (.10), *p* = .010; peer problems β = −.31 (.12), *p* = .012; prosocial β = .27 (.12), *p* = .026, while those at 0 standard deviations were of small effect, conduct problems β = .03 (.07), *p* = .638; peer problems β = −.04 (.07), *p* = .604; prosocial β = .03 (.06), *p* = .635. Hence, stronger relationships between language and conduct, peer problems, and prosocial behavior were observed at lower levels of language. For emotional problems, there was a moderate slope of language proficiency at −1.5 standard deviations, β = −.39 (.12), *p* = .002, which was only marginally different to the slope at 0 standard deviations, β = −.15 (.08), *p* = .046, Wald test = 3.19, 1, *p* = .074. All chi-square tests were significant, but models were uniformly above recommended cutoffs for all other fit indices (Hu & Bentler, 1999). Further nonlinearity was not supported as the addition of a higher-order *L*^3^ cubic term was not predictive of any form of SEB (conduct problems: *p* = .888; peer problems: *p* = .433; emotional problems: *p* = .697; prosocial: *p* = .852).

### Is the Longitudinal Relationship Between Language and SEB Moderated by Sex, NVIQ, or SES?

**Figure 3 fig3:**
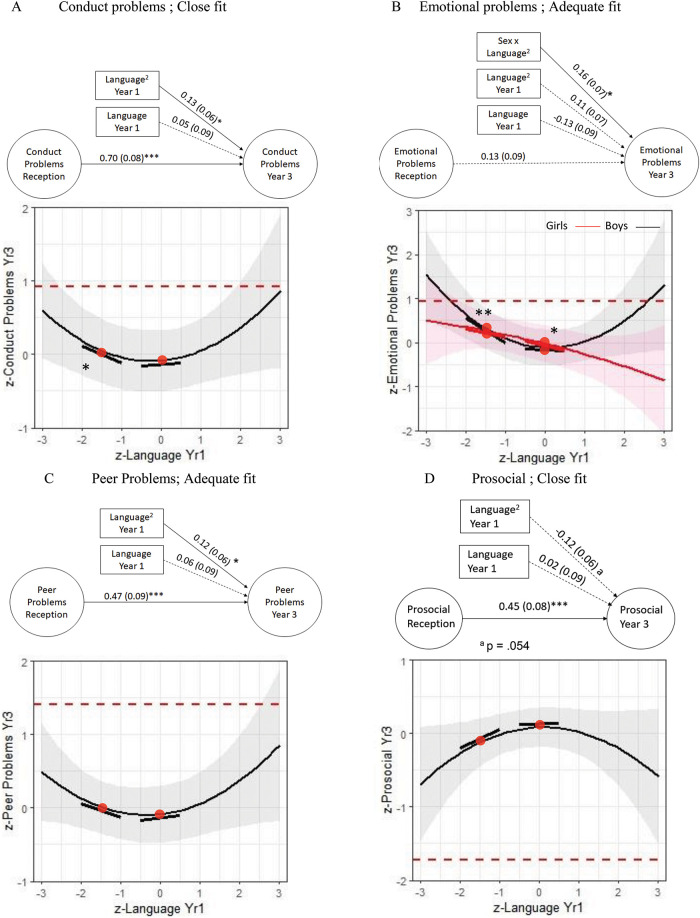
Structural Equation Diagrams Truncated to Only Show Significant Main Effects or Interactions of SES, NVIQ, Sex With Language to (A) Conduct, (B) Emotional, (C) Peer, and (D) Prosocial Problems at Year 3 Adjusted for Prior Levels With Accompanying Decompositions *Note*. Dotted lines denote borderline highest 20% cutoffs. Models *n* = 363, weighted *n* = 6.451. See online Supplemental Materials S6 for nontruncated structural equation diagram with significant and nonsignificant factor loadings and paths of socioeconomic status (SES), nonverbal IQ (NVIQ), and sex. Fit indices of models are (A) χ^2^ = 131.39 (92), *p* = .004, CFI = .97, TLI = .96, RMSEA = .04, 90% CI [.02, .05]; (B) χ^2^ = 146.18 (93), *p* = .000, CFI = .96, TLI = .94, RMSEA = .04, [.03, .05]; (C) χ^2^ = 149.66 (92), *p* = .000, CFI = .96, TLI = .94, RMSEA = .04, [.03, .05]; (D) χ^2^ = 141.83 (93), *p* = .000, CFI = .98, TLI = .98, RMSEA = .04, [.03, .05]. * *p* < .05. ** *p* < .01. *** *p* < .001. See the online article for the color version of this figure.

In the final step, sex, nonverbal ability, and SES were entered as an additional block of moderators to the path models (see Figure 3). Multicollinearity was observed as nonverbal ability and SES were highly correlated with language (e.g., *r* = .70 between Language^2^ and Language × NVIQ; *r* = −.78 between SES × Language and SES × Language^2^), and model results showed standard errors that were unusually large (see online Supplemental Materials S6 for complete models). We therefore pruned the models to only include the main effects of SES and nonverbal ability, while retaining sex as a moderator (see Figure 3).[Fig-anchor fig3]

In these adjusted models in Figure 3, the nonlinear relationship of language to conduct problems was maintained, with a significant slope at −1.5 standard deviations but not at 0 standard deviations of language, −1.5 *SD* β = −.22 (.10), *p* = .030; 0 *SD* β = .05 (.08), *p* = .590. The relationship of Language^2^ to emotional problems was moderated by sex. For boys, language at −1.5 standard deviations but not 0 standard deviations was related to emotional problems, −1.5 *SD* β = −.55 (.19), *p* = .003; 0 *SD* β = −.04 (.12), *p* = .120. For girls, the reverse pattern was found, in which language at 0 standard deviations but not −1.5 standard deviations was related to emotional problems, 0 *SD* β = −.22 (.11), *p* = .040; −1.5 *SD* β = −.17 (.19), *p* = .380. Finally, the nonlinear relationship of Language^2^ to peer problems maintained, while the relationship to prosocial behavior attenuated. Further slope tests indicated a stronger association of language at lower levels of language but were not statistically significant for either peer, 0 *SD* β = .06 (.08), *p* = .480; −1.5 *SD* β = −.19 (.13), *p* = .140, or prosocial outcomes, 0 *SD* β = .02 (.09), *p* = .849; −1.5 *SD* β = .26 (.15), *p* = .079. Main effects of SES and nonverbal ability were not statistically significant and are reported in full in online Supplemental Materials S6. With the exception of the TLI, all other models were uniformly above cutoffs for fit indices (Hu & Bentler, 1999) and were all of at least adequate fit.

## Discussion

We examined three possible sources of variation in the prospective association of language at Year 1 to SEB problems at Year 3 in a longitudinal, population-derived cohort of children with and without language disorders (SCALES; sample *n* = 363; weighted *n* = 6,451). First, we considered language modality as a source of variability by testing a measurement model, which included six standard measures of expressive and receptive language. Second, we examined a threshold account, in which stronger associations with SEB were expected at the tail of the language distribution, with negligible relationships as language approaches the normative mean. Finally, we considered if nonverbal cognition, socioeconomic status, and male sex moderated the relationship between language and SEB. However, collinearity meant only sex could be fully tested as a moderator. To our knowledge, this is the first attempt to empirically verify conditions under which prospective associations may be robustly found between language and SEB.

A single-factor language model was the most parsimonious, had good fit, and was superior to the two-factor expressive-receptive model. This is consistent with a growing literature unable to parse language into distinct domains (Bornstein et al., 2016; Lonigan & Milburn, 2017; Tomblin & Zhang, 2006). We then observed a nonlinear relationship in which SEB did not associate uniformly across the language continuum. Instead, language at the level consistent with diagnosis of DLD (−1.5 standard deviations) had a significant and strong association with conduct, emotional (boys only), peer, and prosocial problems, while the association with language at an average level (0 standard deviations) was not significant. This finding aligns with the larger literature reporting moderately sized increases in levels of SEB among children with DLD (Yew & O’Kearney, 2013) but more variable and much smaller effect sizes among unselected samples with a higher mean level of language (Bornstein et al., 2013; Chow & Wehby, 2018). Thus, variation in initial language levels may account for differences between studies. While our data indicate that language levels in early primary school are important indicators of concurrent and future SEB concern, rate of language growth may also improve prediction of SEB (Westrupp et al., 2019).

Our models (Figures 2 and 3) are suggestive of a U-shaped relationship between language and SEB, in which the contribution of language is amplified at either extreme of the distribution. However, that SEB may be amplified in children with highest language competence is unexpected, and we note the very small numbers of children with exceptional language skills in our sample, yielding correspondingly wide confidence intervals. Given this finding was not predicted, and that exceptional verbal skills are not a recognized risk factor for SEB problems, we did not test slope differences at +1.5 standard deviations. These findings suggest that better than average language skills are not protective of SEB risk, though replication is needed.

It is possible that thresholds reflect the insensitivity of the SDQ to measure “above-average” SEB competence. The SDQ was designed to characterize abnormal SEB, rather than the full range of SEB skills (see online Supplemental Materials S5 for information curves). In this case, the upper-right end of the U-shaped curve, where children with exceptional language cluster, may be “pulled” down to an L shape, or even further down to a continuous linear descending line when the full SEB spectrum is considered. Thus, possible linear relationships may be obscured by poor measurement of above-average SEB outcomes (Cole et al., 2010).

Instead, at the tail of the language distribution, children are at amplified risk of SEB problems. However, in terms of absolute risk, even at the most extreme levels of language deficit, the 95% confidence intervals did not uniformly cross cutoffs for “borderline” concern of SEB. This differs from meta-analytic findings (e.g., Curtis, 2018; Yew & O’Kearney, 2013) and may reflect the fact that this sample is relatively more affluent and had a lower prevalence of SEB than predicted from existing normative data on the SDQ (e.g., 11.6% and 6.2% of entire sample met 80th percentile borderline cutoff for conduct and emotional problems).

In addition, the relationship between language and SEB was estimated in autoregressive models; prior SEB accounts for between 0.01% and 50.4% of variation in SEB outcomes, likely attenuating the predictive effect of language (Adachi & Willoughby, 2015). Our findings are consistent with previous research showing that children with DLD present with elevated symptom profiles that are often below clinical thresholds (St. Clair et al., 2011). The one exception is hyperactivity-inattention, with half of the DLD group receiving teacher ratings within the clinical range at Year 3.

Further examination of the longitudinal relationship between language and hyperactivity-inattention was hampered by a lack of measurement invariance, indicating that the construct of hyperactivity-inattention was not consistent over time (Putnick & Bornstein, 2016). The fact that our study relied on teacher ratings, which by necessity resulted in different respondents at different points in time, may have exacerbated this issue.

Our final question asked if known correlates of language disorder, namely lower nonverbal ability, male sex, and neighborhood disadvantage, moderated the association between language and SEB. Sex significantly influenced the relationship between language and SEB such that lower levels of language yielded more stark emotional problems for boys but not girls. One earlier study reported that while teenaged girls were more susceptible to emotion problems within the total population, there were no sex differences in the cohort with language disorder (Conti-Ramsden & Botting, 2008). It is therefore possible that sex differences may change with age in this population. Few prior studies have tested sex differences in cohorts that include DLD; thus, these findings require replication. We were not able to directly test nonverbal ability and neighborhood disadvantage as moderators due to multicollinearity. It remains challenging to disentangle unique effects of language on SEB from the effects of these highly correlated variables. Nevertheless, language is arguably more malleable than SES or nonverbal cognition, and therefore intervention studies that target language could elucidate causal mechanisms.

For emotional and conduct problems, elevated risk for children with language disorder was evident even after adjustment for nonverbal ability and neighborhood disadvantage. However, relationships with prosocial and peer problems attenuated when covariates were included, and the contribution of any single predictor fell below statistical significance. This highlights the heterogeneous developmental pathways for individuals with language disorder, in which co-occurring SEB deficits are common but not inevitable (Conti-Ramsden et al., 2019). The variable outcome is likely influenced by additional biological and environmental risk factors and perhaps differences brought about by age (Curtis et al., 2018) not measured here.

In sum, the current study provides strong evidence that the relationship between language and SEB is not linear and that risk of adverse SEB outcome is greatest for those with clinically significant levels of language deficit. Children with typically developing language show fewer SEB symptoms, though good language does not appear to confer additional advantage. DLD is associated with nonverbal deficits and socioeconomic disadvantage, but the specific influence of language maintains even when these additional risks are taken into account. Finally, risks are broadly similar for boys and girls, though the development of emotional symptoms may be sex specific. These findings are consistent with the view that in DLD, children fail to develop the oral language skills necessary for positive SEB adjustment. Such skills may include the ability to identify emotional states of self and others (Griffiths et al., 2020), use verbal strategies to regulate their own emotions and behavior, and use language to build positive social networks (Cole et al., 2010; Durkin & Conti-Ramsden, 2010). Positive outcomes of language-based interventions for SEB (e.g., Curtis et al., 2019) support this hypothesis, though whether such interventions would have positive impacts on children with clinical levels of language disorder remains to be seen.

### Strengths and Limitations

The strengths of this study include the relatively large sample size, the wide variety of language and cognitive abilities within the sample, and multiple measures of language, which has not been possible in other large cohort studies. Measures of language and SEB were obtained independently, reducing single-rater bias, and all models take account of prior levels of SEB. Our study was limited by including few children experiencing pronounced socioeconomic disadvantage and a lower than expected prevalence of SEB. Moreover, the SDQ is not a diagnostic instrument. We chose the SDQ because its psychometric properties are well described and it is consistently used in other population studies, allowing direct comparison of our findings with previous research.

Sensitivity analyses suggested that 0% to 10.2% of cases can be flagged as highly influential. Flagged cases cluster at points along the regression line where data points are sparse, such as at the ends of language continuum or at high SDQ values. The impact of these cases was minimal (online Supplemental Materials S7). These results were based on observed manifest (as opposed to latent) variables without adjustment for measurement error. Sample size may be considered as a limitation of the present examination of a threshold account, despite the use of the largest cohort (weighted *n* = 6,451) to date with multiple measures of language among children with and without language disorder.

Finally, although we were able to consider three key moderators, we were unable to include extensive measures of biological and environmental risk that may have enhanced identification of children most at risk. We are therefore unable to elucidate possible mechanisms linking language disorder and SEB, including whether this represents shared biological causal mechanisms (for example, overlapping genetic risk; Newbury et al., 2019), family history of poor mental health (Conti-Ramsden et al., 2019), or a confluence of developmental cascades in which poor language predisposes children to academic underachievement and associated loss of self-esteem (Tomblin et al., 2000; Westrupp et al., 2019).

### Conclusion

In conclusion, the relationship between language and SEB outcomes is not linear but amplified at clinical levels of language disorder. This may explain seemingly discrepant findings reporting larger associations in DLD samples and more variable associations between language and SEB from unselected community samples. Intervention studies are needed to test causal theories that posit limited oral language as an early barrier to robust development of regulatory processes that foster good mental health. In addition, future cohort studies should investigate multiple risk models that combine biological and environmental factors to yield a more comprehensive picture of developmental pathways to good SEB for children with DLD.

## Supplementary Material

10.1037/abn0000691.supp

## Figures and Tables

**Table 11 tbl1:** Weighted Means, Percentages, and Standard Errors of Omnibus and Planned Comparisons Across Children With DLD, Typical Language, and Language Disorder Associated With a Known Diagnosis

	*M*/% (*SE*)			Omnibus test		Planned comparison	
Sample	DLD	TL	Known diagnosis	Overall *F* (*df*)	Overall *p*	*F* (*df*) betweenDLD and TL	*p* betweenDLD and TL
*N* raw, Weighted *n*, %	91, 487.76, 7.6%	392, 5,803.32, 90.1%	45, 151.27, 2.3%	NA	NA	NA	NA
Age (Months)	71.52 (0.68)	71.77 (0.32)	73.08 (0.67)	1.80 (2, 526)	.166	0.11 (1, 527)	.745
Male	54.3% (9.0%)	49.2% (3.3%)	76.8% (7.7%)	113.84 (1.98, 113.84)	**.000**	199.82 (1, 527)	**.000**
NVIQ	−0.77 (0.11)	0.11 (0.06)	−1.82 (0.21)	57.49 (2, 526)	**.000**	50.07 (1, 527)	**.000**
SES deprivation	0.19 (0.02)	0.09 (0.05)	0.15 (0.02)	9.98 (2, 526)	**.000**	15.67 (1, 527)	**.000**
Reception year (4−5 years)							
SDQ CP	0.91 (0.20)	0.73 (0.08)	1.97 (0.41)	4.64 (2, 526)	**.010**	0.70 (1, 527)	.402
SDQ EP	1.07 (0.23)	1.19 (0.11)	2.14 (0.45)	2.33 (2, 526)	.099	0.22 (1, 527)	.637
SDQ HI	4.04 (0.40)	2.20 (0.18)	6.64 (1.20)	14.70 (2, 526)	**.000**	17.74 (1, 527)	**.000**
SDQ PP	1.26 (0.23)	1.12 (0.11)	3.45 (0.58)	7.88 (2, 526)	**.000**	0.29 (1, 527)	.588
SDQ PS	7.25 (0.42)	7.81 (0.16)	4.72 (0.77)	8.17 (2, 526)	**.000**	1.57 (1, 527)	.211
SDQ CP % borderline+	11.8% (3.2%)	10.2% (1.9%)	43.5% (13.4%)	9.21 (1.80, 950.53)	**.000**	0.19 (1, 527)	.666
SDQ EP % borderline+	5.3% (1.9%)	6.4% (1.5%)	11.7% (5.2%)	1.93 (1.93, 1,018.07)	.421	0.22 (1, 527)	.637
SDQ HI % borderline+	23.0% (5.0%)	13.8% (2.1%)	67.8% (12.6%)	17.32 (1.94, 1,023.32)	**.000**	3.62 (1, 527)	.058
SDQ PP % borderline+	26.3% (6.2%)	21.3% (2.6%)	63.2% (12.6%)	7.02 (2.00, 1,049.62)	**.001**	0.60 (1, 527)	.438
SDQ PS % borderline+	26.3% (6.2%)	21.3% (2.6%)	63.2% (12.2%)	7.02 (2.00, 10,049.62)	**.001**	0.60 (1, 527)	.438
Years 3 (7–9 years)							
SDQ CP^a^	1.28 (0.33)	0.64 (0.10)	1.19 (0.47)	2.31 (2, 361)	.100	3.52 (1, 362)	.061
SDQ EP^a^	2.06 (0.39)	1.34 (0.15)	2.93 (0.38)	8.42 (2, 361)	**.000**	2.97 (1, 362)	.086
SDQ HI^a^	4.51 (0.62)	2.47 (0.22)	6.77 (0.91)	14.29 (2, 361)	**.000**	9.78 (1, 362)	**.002**
SDQ PP^a^	1.85 (0.30)	0.97 (0.12)	3.29 (0.43)	15.90 (2, 361)	**.000**	7.50 (1, 362)	**.006**
SDQ PS^a^	6.74 (0.57)	8.07 (0.16)	5.55 (0.33)	24.60 (2, 361)	**.000**	5.18 (1, 362)	**.023**
SDQ CP % borderline+^a^	16.1% (6.6%)	8.4% (2.1%)	19.9% (8.3%)	2.04 (1.70, 615.69)	.139	1.81 (1, 331)	.179
SDQ EP % borderline+^a^	13.9% (6.4%)	9.9% (2.3%)	18.4% (8.0%)	0.69 (1.68, 609.55)	.476	0.42 (1, 331)	.518
SDQ HI % borderline+^a^	49.4% (12.3%)	15.4% (2.8%)	63.4% (16.8%)	10.75 (1.90, 688.35)	**.000**	11.67 (1, 331)	**.001**
SDQ PP % borderline+^a^	15.4% (6.5%)	10.6% (2.4%)	54.4% (14.8%)	8.25 (1.96, 711.72)	.**000**	0.58 (1, 331)	.446
SDQ PS % borderline+^a^	29.3% (9.2%)	13.9% (2.6%)	68.6% (11.3%)	12.10 (1.75, 634.99)	**.000**	3.88 (1, 331)	.050
*Note*. *SE* = standard error; DLD = developmental language disorder; TL = typical language; age = age in months at Year-1 language assessment; NVIQ = nonverbal IQ; SES = socioeconomic status; SDQ = Strengths and Difficulties Questionnaire; CP = conduct problems; CP borderline+ = score > 3; EP = emotional problems; EP borderline+ = score > 4; HI = hyperactivity-inattention; HI borderline+ = score > 6; PP = peer problems; PP borderline score+ = >3; PS = prosocial; PS borderline+ = score > 5. Bold denotes *p*-values < .05.							
^a^ Weighted from *n* = 363 with complete data at Year 3.
