# Supplementary material for: Sources of Variability in the Prospective Relation of Language to Social, Emotional, and Behavior Problem Symptoms: Implications for Developmental Language Disorder
Source: J Abnorm Psychol. 2021 Aug;130(6):676–89. doi: 10.1037/abn0000691 (PMC8459610; doi:10.1037/abn0000691)
Supplement: Supplementary file 1 [file ABN-2020-1581_Suppl.docx]

Online Supplements S1 to S7

Online Supplement S1: Weighted histograms and correlations of study variables and supplemental ethnicity information

Online Supplement S2: Missing Group Analysis

Online Supplement S3: Confirmatory factor analysis of six language measures

Online Supplement S4: Longitudinal Measurement invariance of Strengths Difficulties Questionnaire

Online Supplement S5: Information Curves of Strengths Difficulties Questionnaire

Online Supplement S6: Path diagram and parameter estimates of full and pruned structural equation models

Online Supplement S7: Examination of cases flagged as influential

Online Supplement S1: Weighted histograms and correlations of study variables


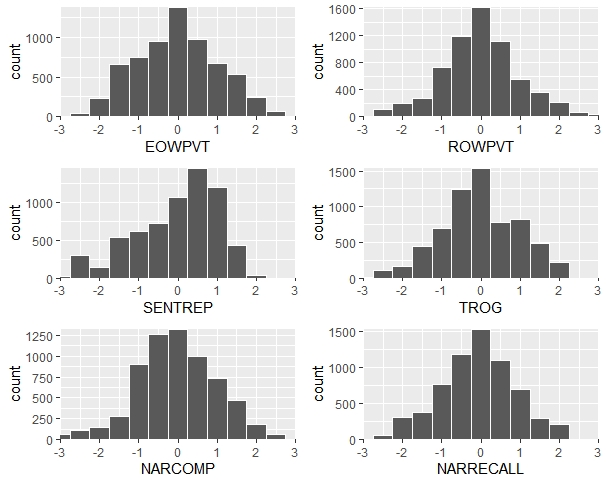


Figure 1. Weighted histograms of six standardised language measures. EOWPVT = Expressive one word picture vocabulary test

Note: ROWPVT = Receptive one word picture vocabulary test; SENTREP = Sentence repetition; TROG = Test of Reception of Grammar; SENTREP = School Age Sentence Repetition Imitation Test; NARCOMP = Narrative Comprehension from the Assessment of Comprehension and Expression ; NARRECALL = Narrative Recall from the Assessment of Comprehension and Expression


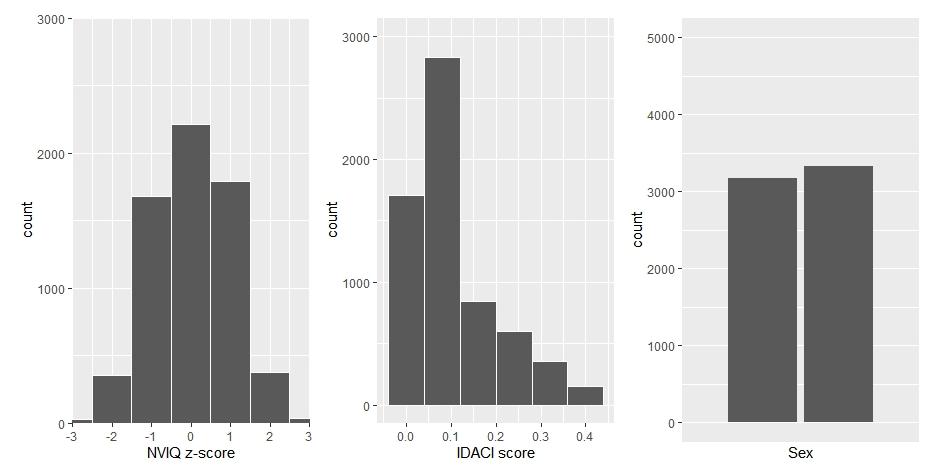


Female Male

Figure 2. Weighted Histograms of proposed moderators of NVIQ, SES (IDACI Score) and Sex. NVIQ = Non-verbal

Note: SES (IDACI Score) = Socio-economic status (Income Deprivation Affecting Children Index Score)


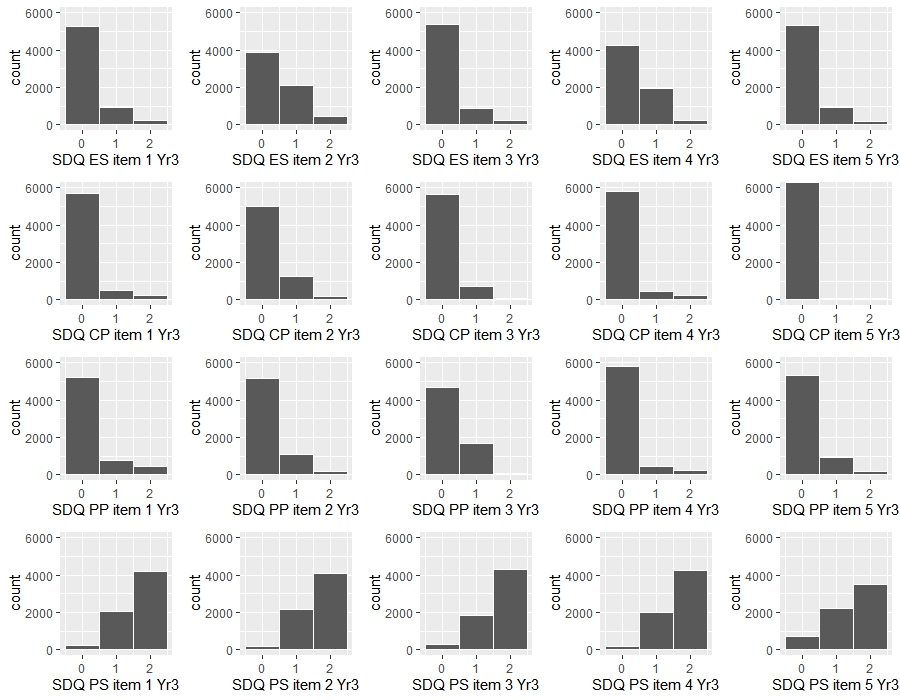


Figure 3. Weighted histograms of Strengths Difficulties Questionnaire for subscales of Emotional, Conduct, Peer and Prosocial Problems

Table 1. Weighted correlation matrix of language, NVIQ, Sex and SES and conduct problem (CP) subscale items at Reception and Year 3 (weighted n = 6,451)

|  | Language Factor Score | Sex | SES | NVIQ | CP1  Reception | CP2  Reception | CP3  Reception | CP4  Reception | CP5  Reception | CP1  Y3 | CP2  Y3 | CP3  Y3 | CP4  Y3 | CP5  Y3 |
| --- | --- | --- | --- | --- | --- | --- | --- | --- | --- | --- | --- | --- | --- | --- |
| Language Factor Score | 1.00 |  |  |  |  |  |  |  |  |  |  |  |  |  |
| Sex | -0.07 | 1.00 |  |  |  |  |  |  |  |  |  |  |  |  |
| SES | -0.35 | 0.03 | 1.00 |  |  |  |  |  |  |  |  |  |  |  |
| NVIQ | 0.57 | 0.07 | -0.21 | 1.00 |  |  |  |  |  |  |  |  |  |  |
| CP1  Reception | -0.03 | 0.16 | 0.02 | 0.01 | 1.00 |  |  |  |  |  |  |  |  |  |
| CP2  Reception | -0.13 | 0.19 | 0.16 | -0.14 | 0.43 | 1.00 |  |  |  |  |  |  |  |  |
| CP3  Reception | -0.02 | 0.17 | 0.03 | -0.04 | 0.38 | 0.49 | 1.00 |  |  |  |  |  |  |  |
| CP4  Reception | -0.06 | 0.20 | 0.03 | 0.00 | 0.46 | 0.44 | 0.40 | 1.00 |  |  |  |  |  |  |
| CP5  Reception | -0.02 | 0.11 | 0.07 | -0.06 | 0.19 | 0.36 | 0.28 | 0.48 | 1.00 |  |  |  |  |  |
| CP1_Y3 | 0.00 | 0.03 | 0.13 | 0.05 | 0.31 | 0.19 | 0.24 | 0.28 | 0.13 | 1.00 |  |  |  |  |
| CP2_Y3 | -0.10 | 0.16 | 0.17 | -0.10 | 0.30 | 0.41 | 0.30 | 0.30 | 0.21 | 0.30 | 1.00 |  |  |  |
| CP3_Y3 | 0.00 | 0.11 | 0.01 | -0.01 | 0.19 | 0.32 | 0.26 | 0.21 | 0.17 | 0.46 | 0.45 | 1.00 |  |  |
| CP4_Y3 | -0.03 | 0.17 | 0.12 | -0.04 | 0.18 | 0.34 | 0.20 | 0.33 | 0.43 | 0.33 | 0.30 | 0.50 | 1.00 |  |
| CP5_Y3 | -0.01 | 0.09 | 0.04 | 0.00 | -0.01 | 0.21 | 0.01 | 0.04 | 0.07 | 0.05 | 0.04 | 0.02 | 0.40 | 1.00 |

Table 2. Weighted correlation matrix of language, NVIQ, Sex and SES and emotional symptoms (ES) subscale items at Reception and Year 3 (weighted n = 6,451)

|  | Language Factor Score | Sex | SES | NVIQ | ES1  Reception | ES2  Reception | ES3  Reception | ES4  Reception | ES5  Reception | ES1  Y3 | ES2  Y3 | ES3  Y3 | ES4  Y3 | ES5  Y3 |
| --- | --- | --- | --- | --- | --- | --- | --- | --- | --- | --- | --- | --- | --- | --- |
| Language Factor Score | 1 |  |  |  |  |  |  |  |  |  |  |  |  |  |
| Sex | -0.07 | 1 |  |  |  |  |  |  |  |  |  |  |  |  |
| SES | -0.35 | 0.03 | 1 |  |  |  |  |  |  |  |  |  |  |  |
| NVIQ | 0.57 | 0.07 | -0.21 | 1 |  |  |  |  |  |  |  |  |  |  |
| ES1  Reception | 0.11 | -0.16 | -0.03 | 0.15 | 1 |  |  |  |  |  |  |  |  |  |
| ES2  Reception | 0.01 | 0.00 | -0.05 | -0.01 | 0.3 | 1 |  |  |  |  |  |  |  |  |
| ES3  Reception | -0.05 | 0.1 | 0.07 | 0.04 | 0.32 | 0.39 | 1 |  |  |  |  |  |  |  |
| ES4  Reception | -0.11 | 0.01 | 0.09 | -0.15 | 0.33 | 0.55 | 0.43 | 1 |  |  |  |  |  |  |
| ES5  Reception | 0.04 | -0.01 | -0.02 | 0.01 | 0.35 | 0.58 | 0.38 | 0.55 | 1 |  |  |  |  |  |
| ES1_Y3 | -0.09 | -0.05 | 0.15 | 0.02 | 0.1 | 0.02 | 0.05 | 0.03 | -0.03 | 1 |  |  |  |  |
| ES2_Y3 | 0.01 | 0.04 | -0.02 | 0.05 | 0.05 | 0.07 | 0.11 | 0.15 | 0.15 | 0.41 | 1 |  |  |  |
| ES3_Y3 | 0.00 | 0.08 | 0.04 | 0.04 | -0.01 | 0.05 | 0.03 | 0.10 | 0.05 | 0.32 | 0.48 | 1 |  |  |
| ES4_Y3 | -0.23 | 0.02 | 0.17 | -0.09 | -0.03 | 0.01 | 0.05 | 0.08 | 0.10 | 0.39 | 0.55 | 0.38 | 1 |  |
| ES5_Y3 | -0.17 | 0.08 | 0.10 | -0.15 | 0.05 | 0.05 | 0.04 | 0.08 | 0.16 | 0.35 | 0.54 | 0.41 | 0.62 | 1 |

Table 3. Weighted correlation matrix of language, NVIQ, Sex and SES and peer problems (PP) subscale items at Reception and Year 3 (weighted n = 6,451)

|  | Language Factor Score | Sex | SES | NVIQ | PP1  Reception | PP2  Reception | PP3  Reception | PP4  Reception | PP5  Reception | PP1 Y3 | PP2 Y3 | PP3 Y3 | PP4 Y3 | PP5 Y3 |
| --- | --- | --- | --- | --- | --- | --- | --- | --- | --- | --- | --- | --- | --- | --- |
| Language Factor Score | 1.00 |  |  |  |  |  |  |  |  |  |  |  |  |  |
| Sex | -0.07 | 1.00 |  |  |  |  |  |  |  |  |  |  |  |  |
| SES | -0.35 | 0.03 | 1.00 |  |  |  |  |  |  |  |  |  |  |  |
| NVIQ | 0.57 | 0.07 | -0.21 | 1.00 |  |  |  |  |  |  |  |  |  |  |
| PP1  Reception | -0.07 | 0.21 | 0.04 | -0.06 | 1.00 |  |  |  |  |  |  |  |  |  |
| PP2  Reception | -0.08 | 0.20 | 0.08 | -0.06 | 0.68 | 1.00 |  |  |  |  |  |  |  |  |
| PP3  Reception | 0.00 | 0.12 | 0.04 | -0.02 | 0.44 | 0.51 | 1.00 |  |  |  |  |  |  |  |
| PP4  Reception | -0.01 | 0.15 | 0.00 | -0.03 | 0.15 | 0.11 | 0.19 | 1.00 |  |  |  |  |  |  |
| PP5  Reception | 0.17 | 0.09 | -0.04 | 0.16 | 0.49 | 0.39 | 0.20 | 0.08 | 1.00 |  |  |  |  |  |
| PP1_Y3 | -0.04 | 0.03 | 0.21 | -0.04 | 0.26 | 0.15 | 0.06 | -0.01 | 0.02 | 1.00 |  |  |  |  |
| PP2_Y3 | -0.14 | 0.13 | 0.09 | -0.14 | 0.31 | 0.29 | 0.24 | 0.11 | 0.07 | 0.32 | 1.00 |  |  |  |
| PP3_Y3 | -0.10 | 0.12 | 0.10 | -0.11 | 0.30 | 0.22 | 0.25 | 0.21 | 0.05 | 0.21 | 0.64 | 1.00 |  |  |
| PP4_Y3 | 0.05 | 0.08 | -0.02 | 0.00 | 0.12 | 0.01 | 0.14 | 0.23 | 0.06 | 0.36 | 0.19 | 0.10 | 1.00 |  |
| PP5_Y3 | 0.12 | 0.14 | 0.11 | 0.12 | 0.24 | 0.20 | 0.18 | 0.17 | 0.16 | 0.35 | 0.32 | 0.33 | 0.09 | 1.00 |

Table 4. Weighted correlation matrix of language, NVIQ, Sex and SES and prosocial subscale (PS) items at Reception and Year 3 (weighted n = 6,451)

|  | Language Factor Score | Sex | SES | NVIQ | PS1  Reception | PS2  Reception | PS3  Reception | PS4  Reception | PS5  Reception | PS1  Y3 | PS2  Y3 | PS3  Y3 | PS4_Y3 | PS5  Y3 |
| --- | --- | --- | --- | --- | --- | --- | --- | --- | --- | --- | --- | --- | --- | --- |
| Language Factor Score | 1.00 |  |  |  |  |  |  |  |  |  |  |  |  |  |
| Sex | -0.07 | 1.00 |  |  |  |  |  |  |  |  |  |  |  |  |
| SES | -0.35 | 0.03 | 1.00 |  |  |  |  |  |  |  |  |  |  |  |
| NVIQ | 0.57 | 0.07 | -0.21 | 1.00 |  |  |  |  |  |  |  |  |  |  |
| PS1  Reception | 0.09 | -0.21 | 0.00 | 0.05 | 1.00 |  |  |  |  |  |  |  |  |  |
| PS2  Reception | 0.11 | -0.15 | -0.07 | 0.14 | 0.71 | 1.00 |  |  |  |  |  |  |  |  |
| PS3  Reception | 0.09 | -0.29 | 0.00 | 0.11 | 0.63 | 0.58 | 1.00 |  |  |  |  |  |  |  |
| PS4  Reception | 0.17 | -0.19 | 0.00 | 0.10 | 0.59 | 0.52 | 0.61 | 1.00 |  |  |  |  |  |  |
| PS5  Reception | 0.22 | -0.24 | -0.09 | 0.15 | 0.60 | 0.52 | 0.67 | 0.59 | 1.00 |  |  |  |  |  |
| PS1_Y3 | 0.10 | -0.15 | -0.16 | 0.11 | 0.31 | 0.24 | 0.34 | 0.24 | 0.25 | 1.00 |  |  |  |  |
| PS2_Y3 | 0.07 | -0.12 | -0.18 | 0.01 | 0.34 | 0.27 | 0.30 | 0.23 | 0.35 | 0.61 | 1.00 |  |  |  |
| PS3_Y3 | 0.08 | -0.19 | 0.02 | 0.04 | 0.33 | 0.22 | 0.33 | 0.18 | 0.33 | 0.58 | 0.44 | 1.00 |  |  |
| PS4_Y3 | 0.09 | -0.10 | 0.00 | 0.05 | 0.20 | 0.12 | 0.22 | 0.14 | 0.15 | 0.45 | 0.44 | 0.61 | 1.00 |  |
| PS5_Y3 | 0.17 | -0.24 | -0.04 | 0.14 | 0.29 | 0.15 | 0.30 | 0.21 | 0.29 | 0.35 | 0.31 | 0.54 | 0.38 | 1.00 |

Table 5. Unweighted ethnicity of the longitudinal cohort of SCALES children at Year I

| Ethnicity | n |
| --- | --- |
| White | 476 |
| Asian or Asian British | 12 |
| Mixed/Dual Background | 24 |
| Any other Ethnic Group (including Black or Black British) | 16 |

Online Supplement S2 : Missing group analysis

Table 1 presents weighted comparisons between these 363 children and the 165 that were not analysed due to missing data. In Year 3, 95% (n = 499) of the in-depth cohort were reassessed (Norbury et al. 2017). Teacher reports of social, emotional and behavioural symptoms using the SDQ (Goodman et al., 1997) were available for 363/499 children (73% of reassessed cohort). These 363 children with SDQ data at Year 3 were analysed in this study and constitute 68.6% of the 528 children profiled at Year 1.

Table 1. Missing group analyses between children with data to be analysed and those who did not

|  | Included in analysis (n = 363) | Not analysed (n= 165) | Overall F (df) | Overall p |
| --- | --- | --- | --- | --- |
| Language Group Status | 7.0% (1.6%) DLD  87.8% (2.1%) TL  5.3% (1.4%) KD | 7.2% (2.2%) DLD  89.2% (2.6%) TL  3.6% (1.4%) KD | .305 (1.98, 1043.95) | .735 |
| Age | 71.69 (0.35) | 71.98 (0.53) | 0.21 (1,527) | .646 |
| Male | 50.1% (3.7%) | 49.2% (5.7%) | 0.02 (1, 527) | .895 |
| NVIQ | 0.02 (0.07) | -0.04 (0.11) | 0.17 (1,527) | .679 |
| SES | 0.99 (0.01) | 0.10 (0.01) | 0.03 (1, 527) | .856 |
| *Reception Year SDQ* | | | | |
| SDQ CP | 0.75 (0.09) | 0.82 (0.14) | 0.19 (1,527) | .666 |
| SDQ EP | 1.16 (0.12) | 1.31 (0.21) | 0.39 (1,527) | .534 |
| SDQ HI | 2.44 (0.21) | 2.47 (0.29) | 0.01 (1,527) | .932 |
| SDQ PP | 1.20 (0.12) | 1.16 (0.19) | 0.04 (1,527) | .845 |
| SDQ PS | 7.81 (0.17) | 7.44 (0.30) | 1.23 (1,527) | .268 |
| SDQ CP % Borderline or above | 11.6% (2.1%) | 10.1% (3.0%) | 0.17 (1,527) | .682 |
| SDQ EP % Borderline or above | 6.2% (1.6%) | 7.1% (2.5%) | 0.11 (1,527) | .744 |
| SDQ HI % Borderline or above | 16.3% (2.4%) | 14.4% (3.3%) | 0.21 (1,527) | .644 |
| SDQ PP % Borderline or above | 22.3% (2.9%) | 23.5% (4.5%) | 0.06 (1,527) | .815 |
| SDQ PS % Borderline or above | 22.3% (2.9%) | 23.5% (4.5%) | 0.06 (1,527) | .815 |

Note : CP=Conduct Problems; DLD= Developmental Language Disorder; EP=Emotional Problems; HI=Hyperactivity-Inattention; KD = Known Diagnosis; PP=Peer Problems; TL = Typical Language

Table 1 weighted results show no difference across study variables. There were no differences between children with or without teacher ratings on age, sex, non-verbal cognition, prevalence of DLD at Year 1, teacher ratings of social, emotional, and behavioural competence at Reception, or socio-economic status as measured by IDACI rank scores, which index neighbourhood deprivation.

Table 2 provides further information on missingness of study variables, by reporting the percentage of missing data.

Table 2. Percentage of missing data on study variables

| Study variable | Missing  (n / % of Year 1 cohort) |
| --- | --- |
| EOWPVT | 0.0 |
| ROWPVT | 0.0 |
| SENTREP | 0.0 |
| TROG | 0.0 |
| NARCOMP | 0.0 |
| NARRECALL | 0.0 |
| Sex | 0.0 |
| NVIQ | 0.0 |
| IDACI | 0.0 |
| Reception SDQ | 0.0 |
| Year 3 SDQ | 165/31.3% |

References

Goodman, R. (1997). The Strengths and Difficulties Questionnaire: a research note. *Journal of Child Psychology and Psychiatry*, *38*(5), 581-586.

## Online Supplement S3 : Confirmatory factor analysis of six language measures
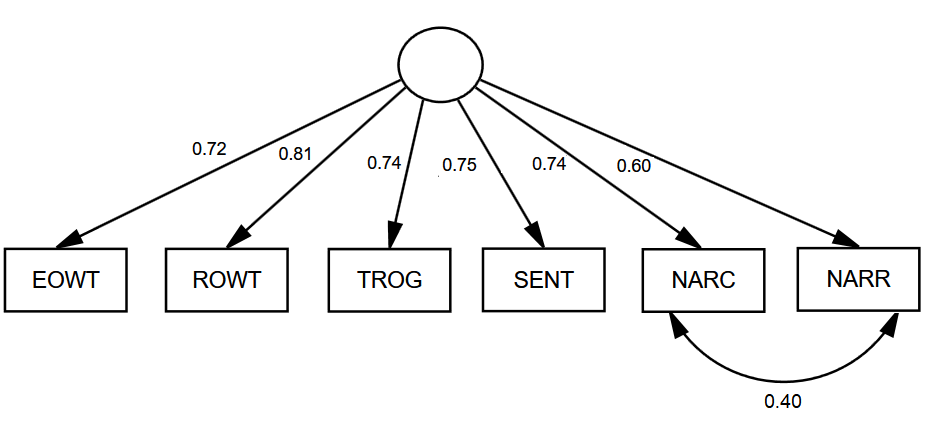

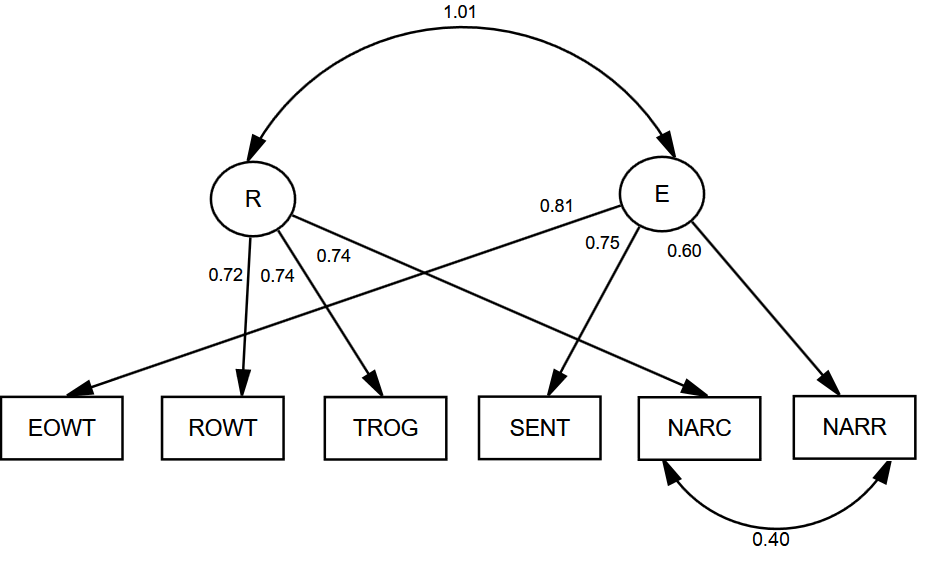


SL

Figure 1. One factor and two factor confirmatory Factor Analysis models of six measures of language.

Note: All factor loadings and correlations are standardised betas that are significantly different from 0 (p<.001). EOWT = Expressive One Word Picture Vocabulary Test; ROWT = Receptive One Word Picture Vocabulary Test; TROG = Test of Reception of Grammar; SENT = School Age Sentence Repetition Imitation Test; NARC = Assessment of Comprehension and Expression Comprehension; NARR = Assessment of Comprehension and Expression Recall; SL = Structural Language; R = Receptive Language, E = Expressive Language.

Figure 1 presents (a) one factor ‘structural language’ (ꭓ^2^=7.37, df = 8, p =.497, CFI = 1.00, TLI = 1.00, RMSEA = .00 [.00,.058], SRMR = 0.02) and (b) two factor ‘expressive-receptive’ confirmatory factor (ꭓ^2^=7.30, df = 7, p =.399, CFI = 1.00, TLI = 1.00, RMSEA = .01 [.00,.066], SRMR = 0.02) models across the six measures of language used in this study. An a priori correlation was specified between narrative recall and comprehension measures as these scores were derived from the same measure (ACE 6 -11; Adams et al., 2001) and thus expected to be more highly related to each other than all other measures of language. Both models showed good fit with non-significant chi-square tests of model fit, though the two factor model contained an inadmissible correlation of 1.01.

The one factor model was named ‘structural language’ consistent with observed factor loadings ranging from 0.60 to 0.81 across all six measures. The two factors in the expressive-receptive model were found to correlate at 1.01. As correlations cannot exceed 1.00, this is an inadmissible parameter, indicating that this model should be dropped. Even if this correlation was to be considered at an admissible number of 1.00, this perfect correlation indicates that these two factors are essentially identical, further supporting the one factor model. For these reasons, the one factor ‘structural language’ model was retained. This latent factor and its factor scores showed high reliability. Here, omega was 0.90, appropriate here as the factor loadings were not all equal, an assumption required for an accurate calculation of Cronbach’s alpha. Moreover, factor score reliability based on Classical Test Theory (Raju, et al., 2007) was also high at 0.90, calculated as the variance of the latent factor of language, divided by the variance of the latent factor of language + standard error of language squared.

Reference

Raju, N. S., Price, L. R., Oshima, T. C., & Nering, M. L. (2007). Standardized conditional SEM: A case for conditional reliability. *Applied Psychological Measurement*, *31*(3), 169-180.

Online Supplement S4 : Longitudinal measurement invariance of Strengths Difficulties Questionnaire


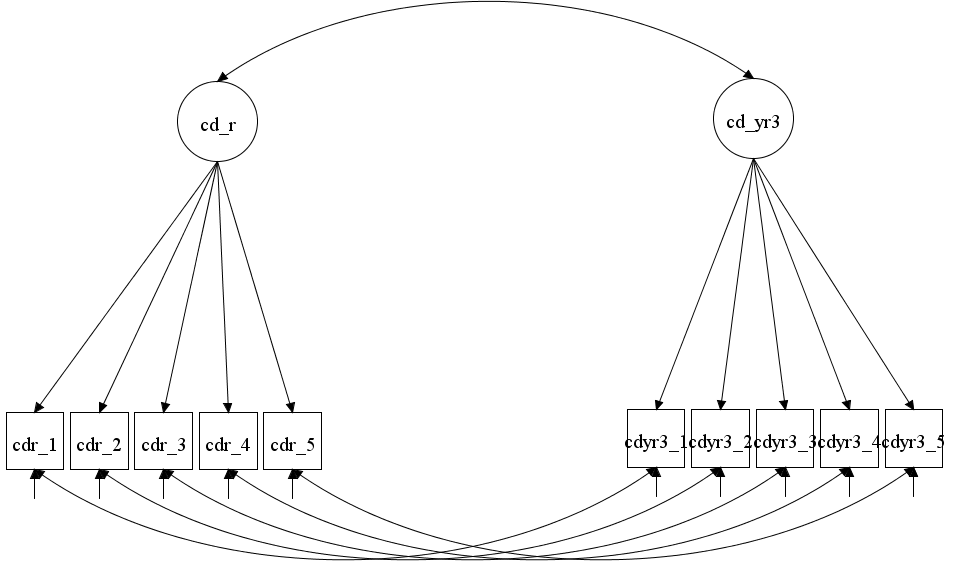

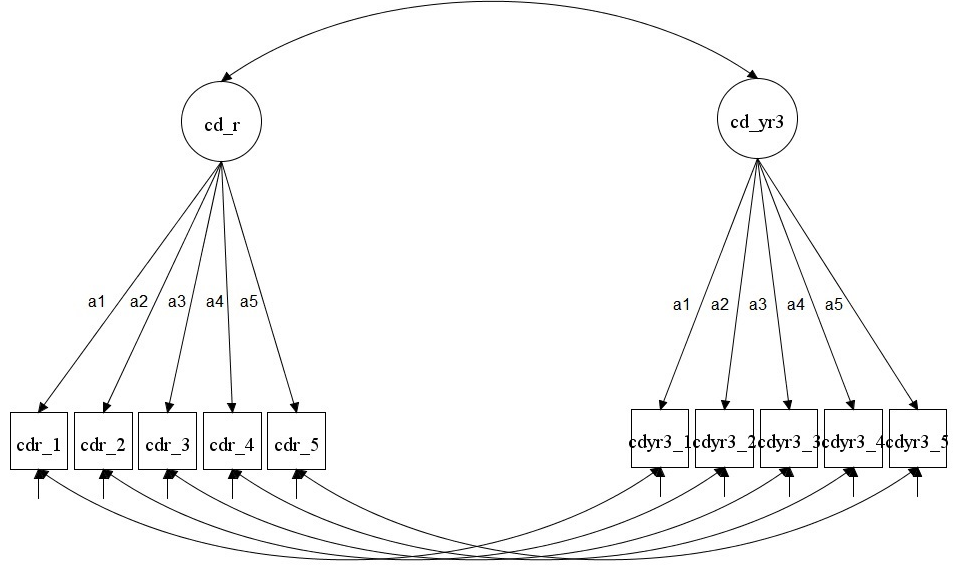

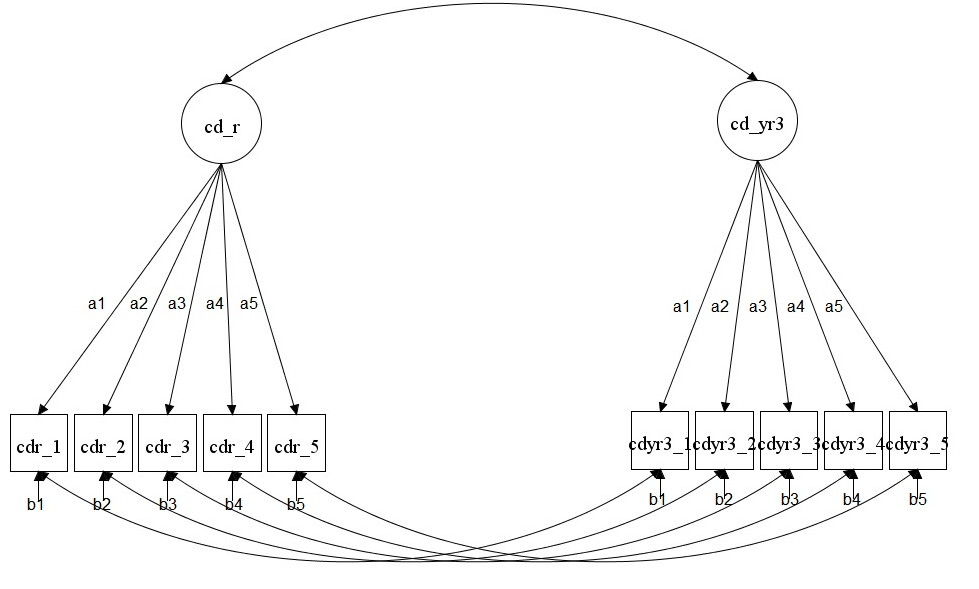


1c

1a 1b

Figure 1. Structural Equation Diagrams of models (1a)Configural invariance (1b)Weak Invariance (1c)Strong Invariance

Figure 1 depicts the three types of structural equation models used to test measurement invariance of socio-emotional behavioural outcomes across two waves of Reception and Year 3. Consistent with the factor structure of the SDQ (Goodman, 1997), a model where all five items were specified to load onto a single factor in each wave was tested.

Table 1. Results of tests of longitudinal measurement invariance of socio-emotional behavioural outcomes across two waves

| Models | Conduct Problems (CD) | Emotional Problems (EP) | Hyperactivity inattention  (HI) | Prosocial  (PS) | Peer Problems (PP) | Chi-square comparison to previous model (ꭓ^2^,df,p) |
| --- | --- | --- | --- | --- | --- | --- |
| 1a: Configural Invariance | Met.  χ2= 73.84, 29, p =.000  CFI = .97, TLI = .95,  RMSEA = 0.07 [0.05, 0.08] | Met  χ2 = 23.87, 29, p = .735  CFI = 1.00, TLI = 1.00,  RMSEA = 0.00  [0.00, 0.03] | Not met.  No convergence. | Met.  χ2= 68.88, 29, p =.000  CFI = .99, TLI = .98,  RMSEA = 0.06 [0.04, 0.08] | Met  χ2= 61.93 ,29, p = .000  CFI = .98, TLI = .96,  RMSEA = .06 [.04, .08] | NA |
| 1b: Weak Invariance : Factor loadings | Partially met^a^  χ2= 75.52, 32, p = .000  CFI = .97, TLI = .95,  RMSEA = .06 [.04, .08] | Met  χ2= 30.46, 33, p = .594  CFI = 1.00, TLI = 1.00, RMSEA = 0.00 [0.00, 0.03] | NA | Partially met.^a^  χ2= 73.08, 32, p =.000  CFI = .99, TLI = .98,  RMSEA = 0.06 [0.04, 0.08] | Partially met^a^  χ2= 67.02, 32, p = .000  CFI = .97, TLI = .96,  RMSEA = .06 [.04, .07] | Model fit does not significantly worsen  CD^a^ = 0.65, 3, p =.885  EP = 7.12, 4, p = .130  PP^a^ = 6.66, 3, p = .083  PS^a^ = 5.69,3, p = .128 |
| 1c: Strong Invariance : Factor loadings and thresholds | Met^a^  χ2= 78.33, 36, p=.000  CFI = 0.97, TLI = 0.96,  RMSEA = .06 [.04, .07] | Met  χ2= 33.65, 37, p = .627. CFI = 1.00, TLI = 1.00,  RMSEA = 0.00  [0.00, 0.03] | NA | Met^a^  χ2= 77.24, 36, p=.000  CFI = 0.99, TLI = 0.98,  RMSEA = .06 [.04, .07]. | Met^a^  χ2= 68.85, 36, p = .001  CFI = 0.98, TLI = 0.97,  RMSEA = .05 [.03, .07] | Model fit does not significantly worsen  CD = 5.51, 4, p =.239  EP = 3.06, 4, p = .548  PP = 2.18, 4, p = .702  PS = 2.68,4, p = .613 |

Note: ^a^Partial invariance was met as factor loading for 1 of 5 items was freed.

As shown in Table 1, for the first type of model ‘1a’, configural invariance, four out of five SEB domains showed configural invariance . The hyperactivity-inattention model failed to converge, suggesting a very poor fit to the data. With the exception of the RMSEA for conduct problems which was 0.01 away from accepted cut-offs for good fit, all other fit indices were within cut-offs for SEB domains of conduct, emotional and peer problems. These three domains were thus judged to be of adequate or very close to adequate fit and retained for further testing. In model 1b weak invariance, these four SEB domains were subsequently found to meet criteria for weak or partial weak invariance. Items here were constrained to the same factor loading across waves (e.g. loading of item1 Reception = loading of item1 Yr3; loading of item 2 Reception = loading of item 2 Yr3, see ‘a1 to a5’ in Figure 1). Emotional problems showed weak invariance, as constraining the factor loadings did not worsen model fit, as shown by a non-significant chi-square model comparison test. Conduct, prosocial and peer problems showed partial weak invariance, as model fit did not worsen when one of five items was allowed to vary across waves.

Finally, as shown in Model 1c Table 1 all four SEB domains met criteria for strong or partial strong invariance (Model 1c). Items here were constrained to the same threshold (see ‘b1 to b5’ in Figure 1) across waves. Emotional problems showed strong invariance, as constraining the intercepts did not worsen model fit. Although constraining the intercepts of conduct, prosocial and peer problems did not worsen model fit, the result from the partial weak invariance is ‘carried forward’ with previously freed factor loading continuing to be freed. Model 1c Emotional problems showed good fit as indicated by a non-significant chi-square test of model fit. Conduct, prosocial and peer problems showed adequate fit, as chi-square tests of model fit were significant while fit indices were above accepted cut-offs (Hu & Bentler, 1999).

Table 2. Parameters constrained to equality across Reception and Year 3 as per model 1c which meets strong or partial strong longitudinal measurement invariance

|  | Conduct | Emotional | Peer | Prosocial |
| --- | --- | --- | --- | --- |
| Factor loadings | | | | |
| Item 1 | X | X | X | X |
| Item 2 | X | X | X | X |

| Item 3^a^ | X | X |  |  |
| --- | --- | --- | --- | --- |
| Item 4 | X | X | X | X |
| Item 5^b^ |  | X | X | X |
| Thresholds | | | | |
| Item 1 | X | X | X | X |
| Item 2 | X | X | X | X |
| Item 3 | X | X | X | X |
| Item 4 | X | X | X | X |
| Item 5 | X | X | X | X |

Note : X Constrained to equality across Reception and Year 3. ^a^Unconstrained items 3 for Peer and Prosocial were questions 14 and 9 on the SDQ respectively. ^b^Unconstrained item 5 Conduct was question 22 on the SDQ.

Online Supplement S5 : Information curves of Strengths Difficulties Questionnaire


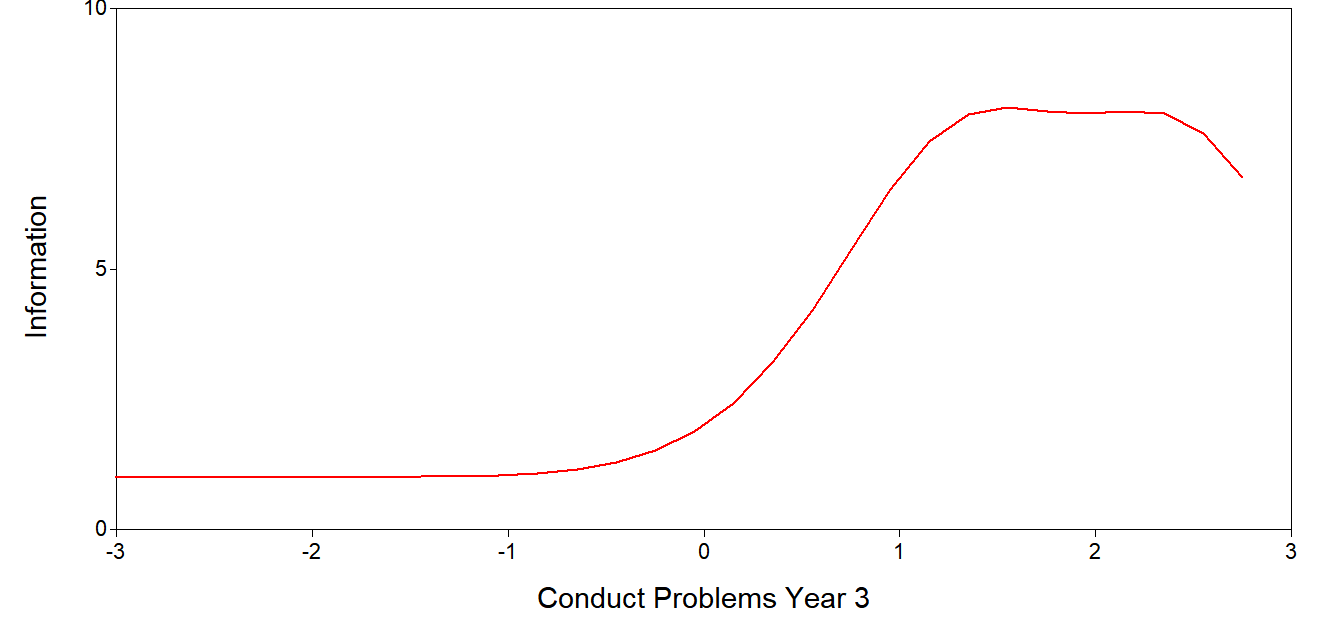


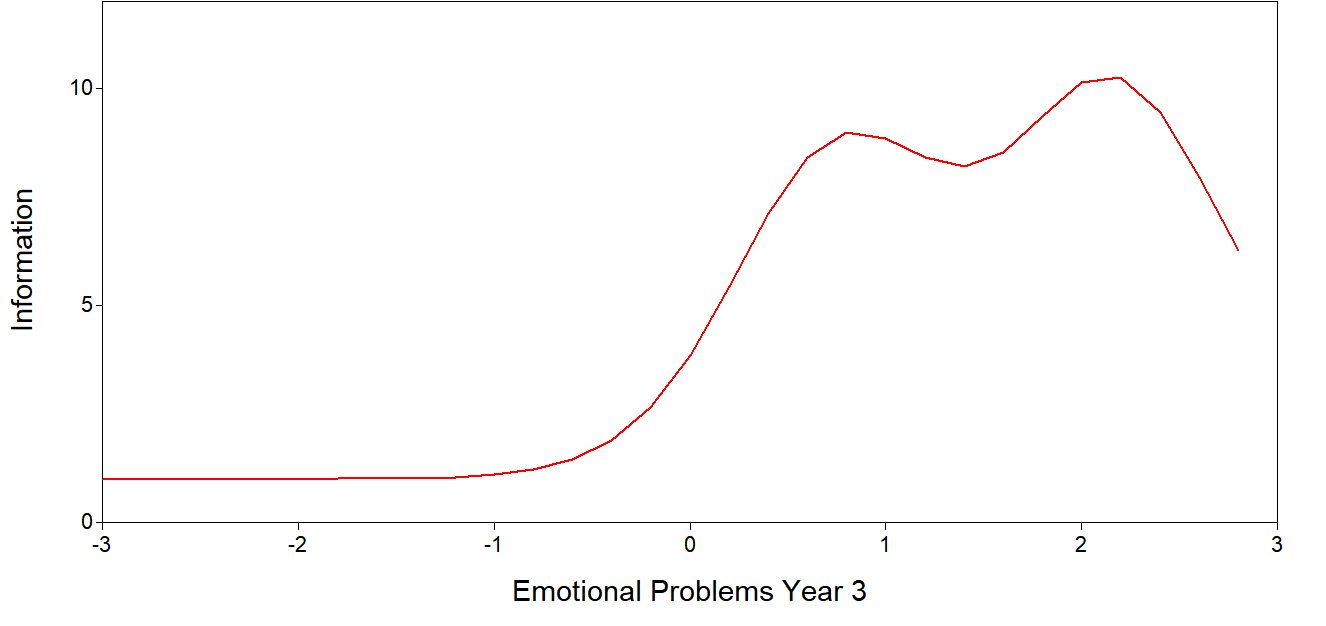


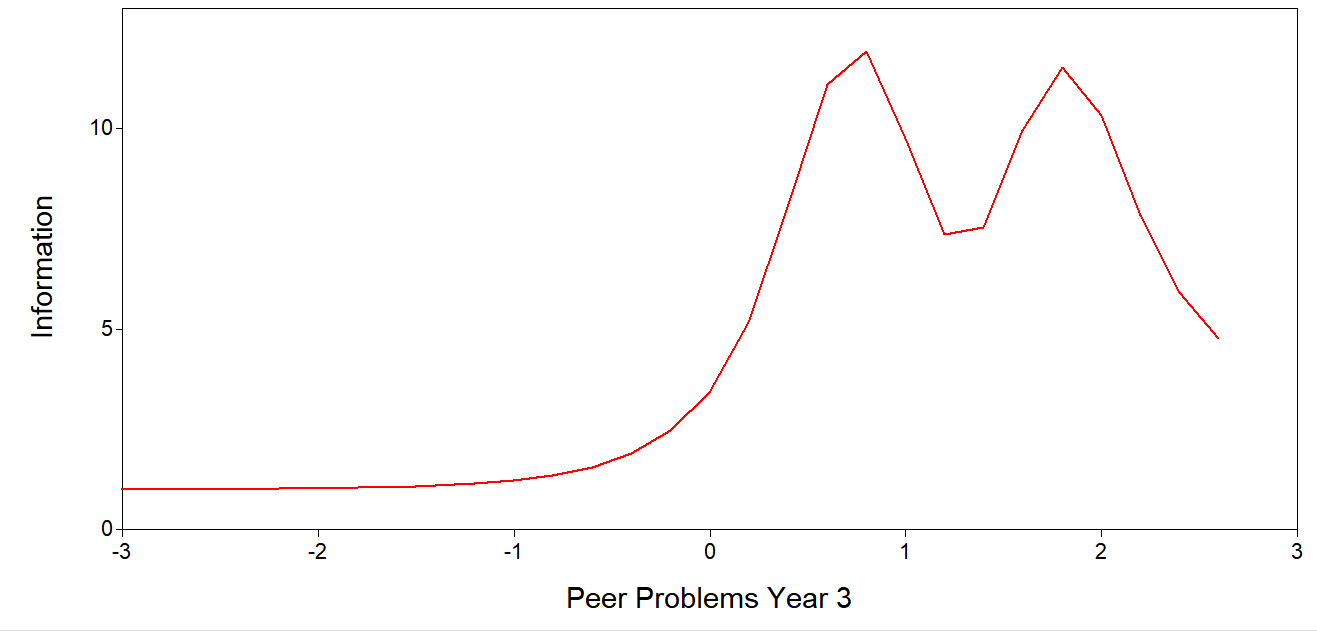


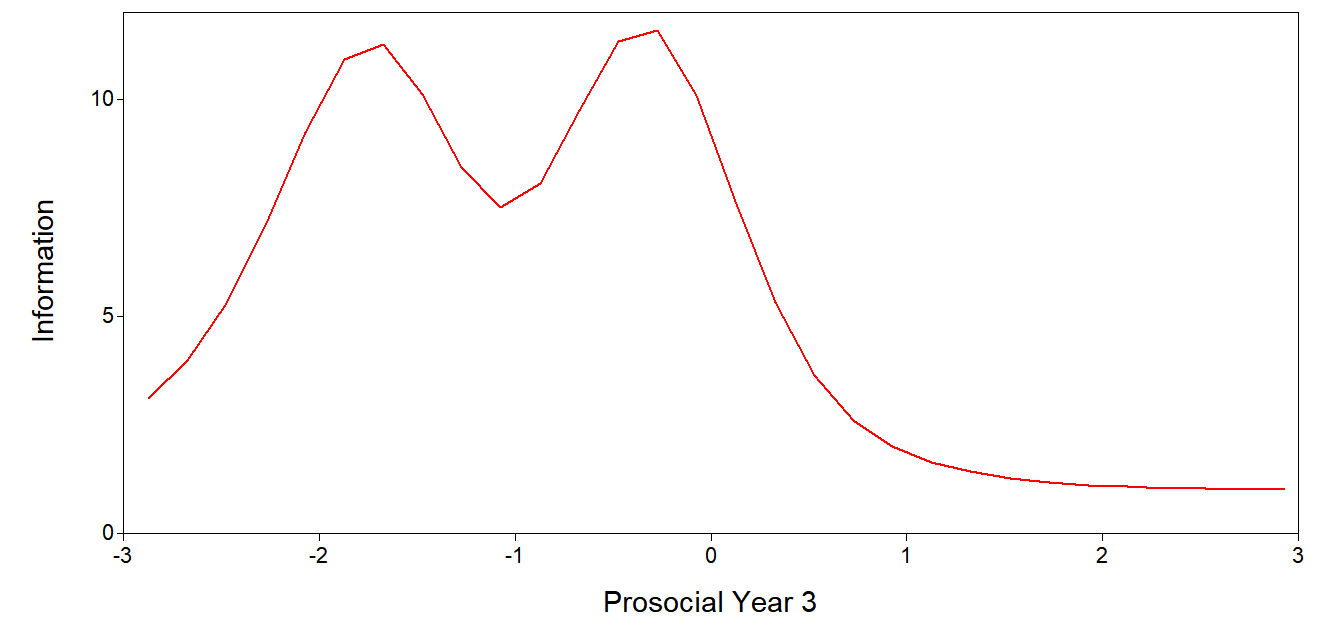


Figure 1. Test information curves for Conduct Problems, Emotional Problems, Peer Problems and Prosocial at Year 3.

Derived from item response theory, the amount of information per each latent construct of conduct, emotional, peer and prosocial are calculated. As shown in Figure 1, tests are informative from 0 to 3 standard deviation levels of the latent construct being estimated. For example, an information of 5, corresponding to a cronbach’s alpha of 0.80 (Demars, 2010), is reached at approximately 0.5 SDs of conduct problems. As the level of conduct problems rises beyond 0.5 SD, the level of information continues to exceed 5 and its corresponding reliability of 0.80. This truncated range of sensitivity, only in the 0 to 3 standard deviation range, is in keeping with the designation of the 5 items of the SDQ conduct scale as a problem subscale (Goodman, 1997). This same pattern is seen for other problem subscales of emotional and peer problems.

For the prosocial scale, the information curve indicates it is sensitive in the range of ‘problems’ of prosociality. Here, an information of 5, corresponding to a cronbach’s alpha of 0.80 (DeMars, 2010), is reached at approximately -2.5 SDs of prosociality. This level of information continues or exceeds 5 and its corresponding reliability of 0.80 until a prosocial level of approximately 0.5 SD. Hence, this scale is not informative at highly skilled or proficient levels of prosociality, but instead measures the ‘problem’ range of prosociality.

References

DeMars, C. (2010). *Item response theory*. Oxford University Press.

Goodman, R. (1997). The Strengths and Difficulties Questionnaire: a research note. *Journal of child psychology and psychiatry*, *38*(5), 581-586.

Online Supplement S6 : Path diagram and parameter estimates of pruned and non-pruned structural equation models


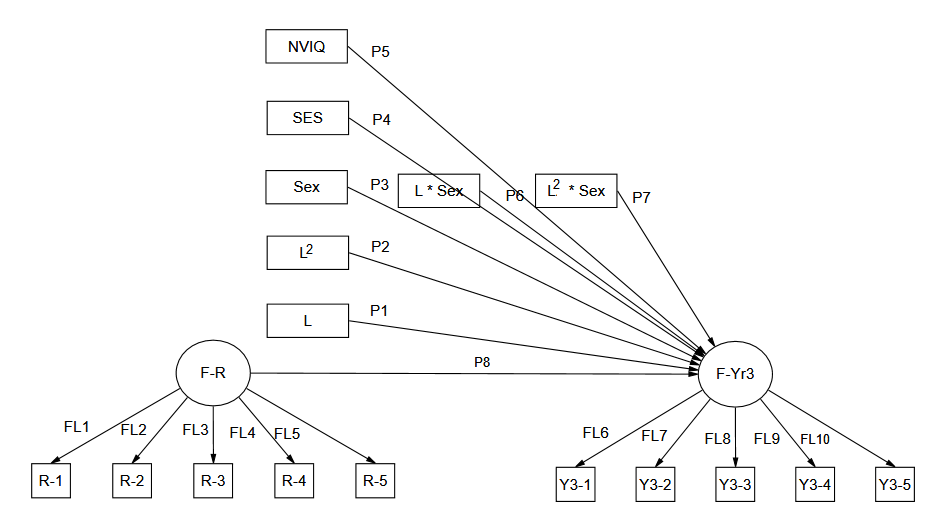


Legend : L = Language factor score Year 1, L^2^ = Language factor score squared, SES = Socioeconomic status, Sex = Sex, NVIQ = Nonverbal Intelligence, F-R = SDQ subscale factor reception, F-Y3 = SDQ subscale factor Year 3.

Figure 1. Structural equation diagram displaying both significant and non-significant main effects and interactions with language to socio-emotional behavioural outcomes at Year 3 adjusted for prior levels. The truncated version of this is displayed in Figure 3 of the main manuscript. See Table 1 below for parameter estimates. Covariance between indicators, predictors and F-R not shown for clarity.

Table 1. Unstandardised and standardised betas of Figure 1 of the contribution of language, SES, gender and NVIQ across SEB outcomes of Conduct Problems, Emotional problems and Peer Problems

|  | Conduct Problems (CP)^a^ | | Emotional Problems (EP)^b^ | | Peer Problems (PP)^c^ | | Prosocial (PS)^d^ | |
| --- | --- | --- | --- | --- | --- | --- | --- | --- |
| Path | Unstandardised Beta (SE) | Standardised Beta (SE) | Unstandardised Beta (SE) | Standardised Beta (SE) | Unstandardised Beta (SE) | Standardised Beta (SE) | Unstandardised Beta (SE) | Standardised Beta (SE) |
| *Paths (P1 to P8)* | | | | | | |  |  |
| 1. L | 0.08 (0.14) | 0.05 (0.09) | -0.16 (0.11) | -0.13 (0.09) | 0.16 (0.23) | 0.06 (0.09) | 0.05 (0.27) | 0.02 (0.09) |
| 1. L^2^ | 0.21 (0.09)* | 0.13 (0.06)* | 0.13 (0.08) | 0.11 (0.07) | 0.32 (0.16)^e^ | 0.12 (0.06)* | -0.33 (0.18)^f^ | -0.12 (0.06)^g^ |
| 1. SES | 1.00 (1.09) | 0.08 (0.08) | 0.72 (0.92) | 0.08 (0.10) | 2.95 (1.90) | 0.14 (0.09) | -0.87 (2.17) | -0.04 (0.09) |
| 1. Sex | 0.20 (0.23) | 0.08 (0.08) | -0.08 (0.19) | -0.05 (0.11) | 0.27 (0.40) | 0.07 (0.10) | -0.72 (0.44) | -0.16 (0.10) |
| 1. NVIQ | 0.02 (0.11) | 0.02 (0.09) | 0.01 (0.07) | 0.02 (0.08) | -0.16 (0.14) | -0.08 (0.07) | 0.00 (0.18) | 0.00 (0.08) |
| 1. L x Sex | 0.22 (0.21) | 0.06 (0.06) | 0.23 (0.17) | 0.09 (0.07) | -0.30 (0.35) | -0.06 (0.06) | 0.27 (0.38) | 0.04 (0.06) |
| 1. L^2^ x Sex | -0.21 (0.18) | -0.08 (0.06) | 0.31 (0.15)* | 0.16 (0.07)* | 0.13 (0.29) | 0.03 (0.07) | 0.45 (0.32) | 0.10 (0.07) |
| 1. F-R | 0.73 (0.15)*** | 0.70 (0.08)*** | 0.13 (0.10) | 0.13 (0.09) | 0.42 (0.11)*** | 0.47 (0.09)*** | 0.42 (0.09)*** | 0.45 (0.08)*** |
| *Factor Loading (FL 1 to FL 10)* | | | | | | |  |  |
| 1 R-1 | 1.00 (0.00) | 0.77 (0.05)*** | 1.00 (0.00) | 0.66 (0.06)*** | 1.00 (0.00) | 0.91 (0.03)*** | 1.00 (0.00) | 0.92 (0.02)*** |
| 2 R-2 | 1.27 (0.29)*** | 0.83 (0.04)*** | 2.22 (0.60)*** | 0.89 (0.04)*** | 1.08 (0.41)** | 0.92 (0.04)*** | 0.68 (0.15)*** | 0.85 (0.03)*** |
| 3 R-3 | 1.02 (0.20)*** | 0.77 (0.04)*** | 1.21 (0.28)*** | 0.73 (0.06)*** | 0.49 (0.10)*** | 0.73 (0.04)*** | 0.89 (0.17)*** | 0.91 (0.02)*** |
| 4 R-4 | 1.78 (0.42)*** | 0.90 (0.03)*** | 1.70 (0.31)*** | 0.83 (0.04)*** | 0.31 (0.09)*** | 0.56 (0.08)*** | 0.65 (0.14)*** | 0.84 (0.03)*** |
| 5 R-5 | 1.49 (0.34)*** | 0.87 (0.04)*** | 2.01 (0.41)*** | 0.87 (0.04)*** | 0.31 (0.08)*** | 0.57 (0.08)*** | 0.75 (0.16)*** | 0.88 (0.03)*** |
| 6 Y3-1 | 1.00 (0.00) | 0.77 (0.06)*** | 1.00 (0.00) | 0.62 (0.07)*** | 1.00 (0.00) | 0.67 (0.07)*** | 1.00 (0.00) | 0.88 (0.03)*** |
| 7 Y3-2 | 1.27 (0.29)*** | 0.79 (0.06)*** | 2.22 (0.60)*** | 0.83 (0.03)*** | 1.08 (0.41)** | 0.94 (0.05)*** | 0.68 (0.15)*** | 0.82 (0.04)*** |
| 8 Y3-3 | 1.02 (0.20)*** | 0.83 (0.04)*** | 1.21 (0.28)*** | 0.75 (0.05)*** | 0.72 (0.18)*** | 0.87 (0.05)*** | 1.11 (0.24)*** | 0.92 (0.03)*** |
| 9 Y3-4 | 1.78 (0.42)*** | 0.85 (0.06)*** | 1.70 (0.31)*** | 0.90 (0.03)*** | 0.31 (0.09)*** | 0.42 (0.06)*** | 0.65 (0.14)*** | 0.73 (0.04)*** |
| 10 Y3-5 | 0.62 (0.23)*** | 0.39 (0.08)*** | 2.01 (0.41)*** | 0.94 (0.04)*** | 0.31 (0.08)*** | 0.65 (0.07)*** | 0.75 (0.16)*** | 0.68 (0.05)*** |

Note: ^a^Partial strong invariance constrains applied to CP reception and Yr 3. ^b^Strong invariance constrains applied to EP reception and Yr 3. ^c^Partial strong invariance constrains applied to PP reception and Yr 3. ^d^Partial strong invariance constrains applied to PS reception and Yr 3. ^e^p =.053. ^f^p =.063, ^g^p = .054.


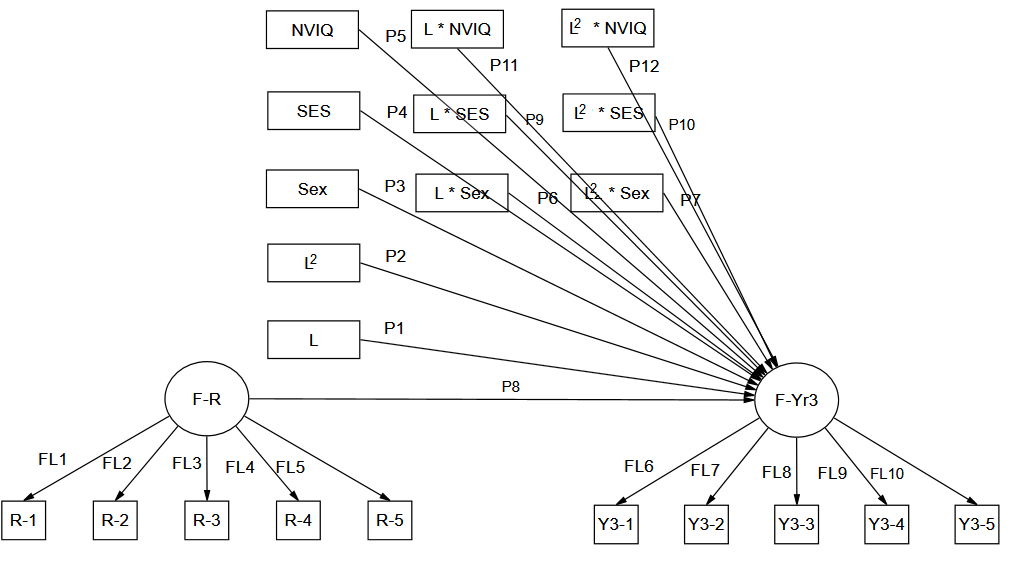


Legend : L = Language factor score Year 1, L^2^ = Language factor score squared, SES = Socioeconomic status, Sex = Sex, NVIQ = Nonverbal Intelligence, F-R = SDQ subscale factor reception, F-Y3 = SDQ subscale factor Year 3.

Figure 2. Non-pruned multicollinear structural equation diagram of both significant and non-significant main effects and interactions with language to socio-emotional behavioural outcomes at Year 3 adjusted for prior levels. See Table 2 below for parameter estimates. Covariance between indicators, predictors and F-R not shown for clarity.

Table 2. Unstandardised and standardised betas from Figure 2 of the contribution of language, SES, gender and NVIQ across outcomes of Conduct Problems, Emotional problems and Peer Problems

|  | Conduct Problems (CP)^a^ | | Emotional Problems (EP)^b^ | | Peer Problems (PP)^c^ | | Prosocial (PS)^d^ | |
| --- | --- | --- | --- | --- | --- | --- | --- | --- |
| Path | Unstandardised  Beta (SE) | Standardised  Beta (SE) | Unstandardised Beta (SE) | Standardised Beta (SE) | Unstandardised Beta (SE) | Standardised Beta (SE) | Unstandardised Beta (SE) | Standardised Beta (SE) |
| *Paths (P1 to P12)* | | | | | | |  |  |
| 1. L | 0.19 (0.16) | 0.11 (0.09) | -0.18 (0.14) | -0.15 (0.11) | 0.16 (0.29) | 0.06 (0.11) | 0.01 (0.32) | 0.00 (0.11) |
| 1. L^2^ | 0.71 (0.18)*** | 0.43 (0.09)*** | 0.23 (0.12) | 0.21 (0.10)* | 0.47 (0.28) | 0.19 (0.11) | -0.49 (0.31) | -0.17 (0.11) |
| 1. SES | 1.54 (1.44) | 0.12 (0.11) | 1.81 (1.19) | 0.20 (0.12) | 3.05 (2.12) | 0.16 (0.11) | -2.55 (2.64) | -0.11 (0.12) |
| 1. Sex | 0.18 (0.24) | 0.07 (0.10) | -0.06 (0.19) | -0.03 (0.11) | 0.26 (0.37) | 0.07 (0.10) | -0.79 (0.43) | -0.18 (0.09) |
| 1. NVIQ | 0.04 (0.12) | 0.03 (0.10) | 0.03 (0.08) | 0.03 (0.10) | -0.14 (0.14) | -0.07 (0.07) | -0.03 (0.19) | -0.01 (0.09) |
| 1. L x Sex | 0.54 (0.25)* | 0.16 (0.07)* | 0.30 (0.19) | 0.20 (0.23) | -0.15 (0.33) | -0.03 (0.06) | 0.15 (0.42) | 0.02 (0.07) |
| 1. L^2^ x Sex | -0.02 (0.18) | -0.01 (0.07) | 0.21 (0.16) | -0.10 (0.25) | 0.18 (0.29) | 0.05 (0.07) | 0.57 (0.37) | 0.12 (0.08) |
| 1. F-R | 0.74 (0.15)*** | 0.72 (0.07)*** | 0.13 (0.10) | 0.13 (0.09) | 0.40 (0.11)*** | 0.46 (0.10) | 0.40 (0.09)*** | 0.43 (0.08)*** |
| **9 L x SES** | 3.92 (2.87) | 0.27 (0.19) | 2.04 (2.34) | 0.20 (0.23) | 0.30 (5.33) | 0.01 (0.24) | -5.78 (5.40) | -0.23 (0.21) |
| **10 L^2^ x SES** | 1.28 (2.04) | 0.14 (0.22) | -0.62 (1.59) | -0.10 (0.25) | -0.28 (2.85) | -0.02 (0.21) | -0.55 (3.03) | -0.04 (0.19) |
| **11 L x NVIQ** | -0.47 (0.16)** | -0.33 (0.09)*** | 0.03 (0.07) | 0.03 (0.07) | -0.20 (0.22) | -0.09 (0.10) | -0.14 (0.26) | -0.05 (0.10) |
| **12 L^2^ x NVIQ** | -0.04 (0.07) | -0.05 (0.09) | -0.01 (0.05) | -0.02 (0.09) | -0.03 (0.10) | -0.03 (0.08) | 0.02 (0.12) | 0.01 (0.09) |
| *Factor Loading (FL 1 to FL 10)* | | | | | | |  |  |
| 1 R-1 | 1.00 (0.00) | 0.77 (0.05)*** | 1.00 (0.00) | 0.65 (0.06)*** | 1.00 (0.00) | 0.91 (0.03)*** | 1.00 (0.00) | 0.92 (0.02)*** |
| 2 R-2 | 1.24 (0.24)*** | 0.83 (0.04)*** | 2.24 (0.59)*** | 0.89 (0.04)*** | 1.10 (0.42)** | 0.92 (0.04)*** | 0.69 (0.15)*** | 0.85 (0.03)*** |
| 3 R-3 | 1.00 (0.19)*** | 0.77 (0.04)*** | 1.20 (0.27)*** | 0.72 (0.06)*** | 0.48 (0.10)*** | 0.72 (0.05)*** | 0.90 (0.17)*** | 0.90 (0.02)*** |
| 4 R-4 | 1.76 (0.41)*** | 0.91 (0.03)*** | 1.79 (0.34)*** | 0.84 (0.04)*** | 0.29 (0.09)** | 0.53 (0.08)*** | 0.66 (0.14)*** | 0.84 (0.03)*** |
| 5 R-5 | 1.43 (0.32)*** | 0.87 (0.04)*** | 2.06 (0.43)*** | 0.87 (0.04)*** | 0.39 (0.10)*** | 0.64 (0.07)*** | 0.78 (0.16)*** | 0.88 (0.03)*** |
| 6 Y3-1 | 1.00 (0.00) | 0.77 (0.06)*** | 1.00 (0.00) | 0.60 (0.07)*** | 1.00 (0.00) | 0.66 (0.07)*** | 1.00 (0.00) | 0.88 (0.04)*** |
| 7 Y3-2 | 1.24 (0.24)*** | 0.80 (0.06)*** | 2.24 (0.59)*** | 0.82 (0.03)*** | 1.10 (0.42)** | 0.93 (0.05)*** | 0.69 (0.15)*** | 0.82 (0.04)*** |
| 8 Y3-3 | 1.00 (0.19)*** | 0.83 (0.04)*** | 1.20 (0.27)*** | 0.73 (0.05)*** | 0.77 (0.19)*** | 0.89 (0.05)*** | 1.12 (0.23)*** | 0.91 (0.03)*** |
| 9 Y3-4 | 1.76 (0.41)*** | 0.85 (0.06)*** | 1.79 (0.34)*** | 0.92 (0.03)*** | 0.29 (0.09)** | 0.39 (0.06)*** | 0.66 (0.14)*** | 0.73 (0.04)*** |
| 10 Y3-5 | 0.60 (0.22)** | 0.39 (0.08)*** | 2.06 (0.43)*** | 0.93 (0.04)*** | 0.39 (0.10)*** | 0.67 (0.07)*** | 0.78 (0.16)*** | 0.70 (0.05)*** |

Note : Pruned paths bolded. Note: ^a^Partial strong invariance constrains applied to CP reception and Yr 3. ^b^Strong invariance constrains applied to EP reception and Yr 3. ^c^Partial strong invariance constrains applied to PP reception and Yr 3. ^d^Partial strong invariance constrains applied to PS reception and Yr 3.


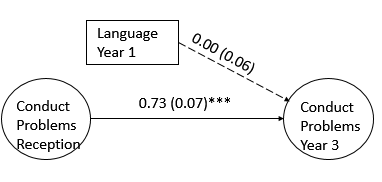

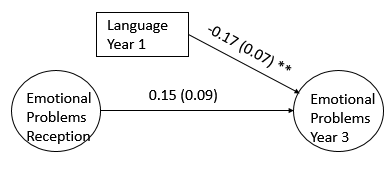


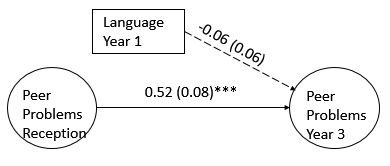

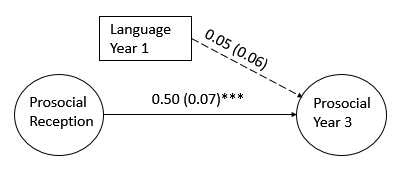


Figure 3. Path models of Year 1 language predicting two-wave lagged socio-emotional behavioural problems

Online Supplement S7 : Examination of cases flagged as influential

**Analytic Strategy**

In addition to existing model fit indices available in the main manuscript, further examination of cases flagged as influential was conducted here. An observed variable approach was utilised here as the WLSMV latent variable approach in the main manuscript is unable to quantify data points of undue influence or large regression residuals. In keeping with the latent variable models in the main manuscript, two sets of regression models were run. This includes (i) the relationship of language to SEB, adjusting for prior SEB and (ii) the relationship of language to SEB with known predictors of sex, NVIQ and SES, adjusting for prior SEB.

Influential case analysis for weighted data can be considered to be an area of ongoing research, with implementation in the R package ‘svydiagnostics’ which we utilise here. Standardised residuals and Cooks D taking into account weights calculated, where values of >3 and >2 respectively (Li & Valliant, 2015; Valliant & Valliant, 2018) were flagged as possible outliers. Linear regressions were calculated in the R package ‘survey’, with weights which adjust for design features and missing data incorporated with the ‘svydesign’ command.

**Results**

*Question 1 : Do influential cases impact the relationship of language to SEB adjusting for prior SEB : Weighted Regression models 1a to 1d*

To assist in visualisation of influential cases, Figure 1 plots naïve unweighted scatterplots of Models 1a, 1b, 1c and 1d. These do not account for weights or the additional covariate of prior SDQ score at Reception, which simply cannot be incorporated into a single plot.

Points coloured red have a standardised residual >3 and can be observed to be far away from the naïve quadratic curve plot. This is consistent with high standardised residuals which indicate a large difference between the predicted and observed point.

Points coloured blue have a Cooks D >2, which suggests that these data points when removed will have a particularly strong influence on regression beta estimates. It appears that these points appear in regions where there are a relatively low density of points. This is adjusted for in the main analysis by sample weights, which assign a larger weight to these points. Recall that these naïve plots do not represent these weighted estimates.

A B


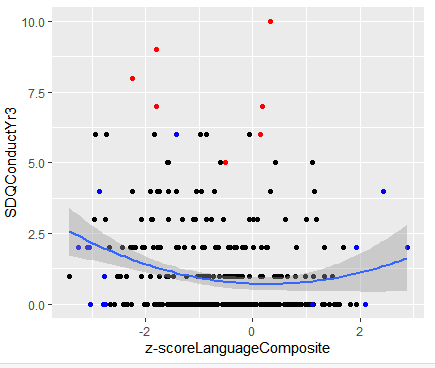

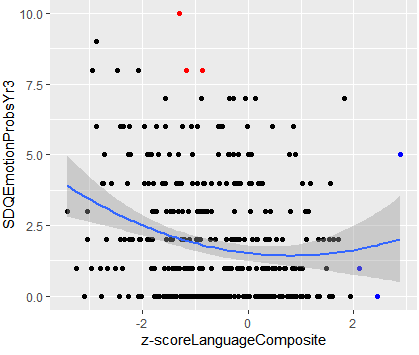


C D


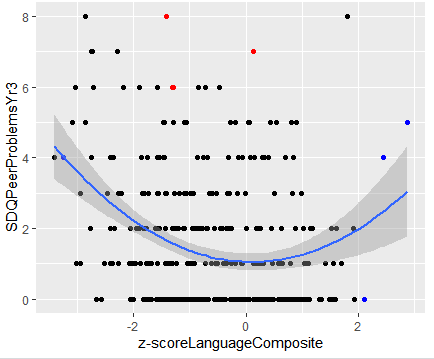

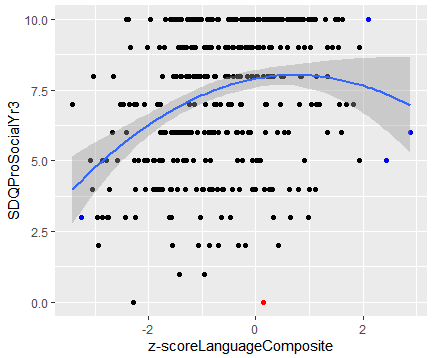


Figure 1. Plots of naïve unweighted scatterplots of a) conduct problems, b) emotional problems, c) peer problems and d) prosocial.

Further sensitivity analysis was conducted to consider the impact of removing these flagged cases. As shown in Table 1, flagged cases made up 1.4 to 6.8% of all cases depending on SDQ outcome. After flagged cases were removed, re-estimated emotional and prosocial models were generally free of flagged cases, but these continued to be present in conduct and peer problem models. The significance of the betaweights across all four models were similar (i.e. significant remained significant) before and after the removal of these flagged cases. Change in p-values was minimal as 1 of 9 parameters changed, with the language squared term for the peer problems dropping to a marginal level of p= .064.

Table 1. Sensitivity analysis of regression models utilising observed variables with and without influential cases

|  |  | Model 1a:  Conduct Problems | Model 1b: Emotional Problems | Model 1c:  Peer Problems | Model 1d: Prosocial |
| --- | --- | --- | --- | --- | --- |
| *Multiple regression models* | | | | | |
| Predictors (Unstd b, SE, p-value) |  |  |  |  |  |
| Language composite |  | -0.13 (0.09)  P = .142 | -0.40 (0.15)  P = **.008** | -0.26 (0.11)  P = **.024** | 0.41 (0.14)  P = **.004** |
| Language composite squared |  | 0.11 (0.05)  P= **.037** | 0.16 (0.10)  P = .108 | 0.20 (0.07)  P = **.003** | -0.21 (0.07)  P= **.004** |
| Reception Year SDQ |  | 0.45 (0.09)  P = **.000** | 0.12 (0.08)  P = .105 | 0.29 (0.08)  P = **.000** | -0.34 (0.06)  P = **.000** |
| Model (sample sizes) |  |  |  |  |  |
| model n; weighted n |  | 363;  6489 | 363;  6489 | 363;  6489 | 363;  6489 |
| n. Cooks d >2 |  | 14 | 3 | 4 | 4 |
| n. Standardised residuals >+/-3 |  | 7 | 3 | 4 | 1 |
| Total n. influential cases |  | 21 (5.8%) | 6 (1.7%) | 8 (2.2%) | 5 (1.4%) |
| *Multiple regression models with influential cases removed* | | | | | |
| Predictors (Unstd b, SE, p-value) |  |  |  |  |  |
| Language composite |  | -0.10 (0.09)  P = .271 | -0.36 (0.14)  P = **.010** | -0.22 (0.11)  P = **.036** | 0.41(0.14)  P = **.003** |
| Language composite squared |  | 0.10 (0.05)  P = **.049** | 0.12 (0.11)  P = .258 | 0.13 (0.07)  P = .064 | -0.22 (0.09)  P = **.014** |
| Reception Year SDQ |  | 0.44 (0.09)  P = **.000** | 0.12 (0.08)  P = .100 | 0.28 (0.08)  P = **.000** | -0.34 (0.06)  P = **.000** |
| Model (Sample sizes) |  |  |  |  |  |
| Sample n; weighted n |  | 342  ;6196 | 357;  6333 | 355;  6327 | 358  ;6340 |
| n. Cooks d >2 |  | 14 | 0 | 6 | 1 |
| n. Standardised residuals >+/-3 |  | 5 | 1 | 1 | 0 |
| Total n. influential cases |  | 19 (5.6%) | 1 (0.3%) | 7 (2.0%) | 1 (0.3%) |

# p = .064

*Question 2 : Do influential cases impact the relationship of language to SEB with known predictors of sex, NVIQ and SES, adjusting for prior SEB : Weighted Regression models 2a to 2d*
A visual scatterplot such as Figure 1 depicting the unweighted relationship between two variables of language and SDQ outcome could not be provided here, as it is more likely to deviate from the actual regression models due to the substantial increase in number of predictors from 3 to 8.

Table 2 shows sensitivity analysis before and after removal of flagged cases 1.3 to 9.9 % of the sample. The significance of the betaweights across all four models were largely similar (i.e. significant remained significant). 3 out of 9 parameters changed in significance. In the peer problems model, the (i) language composite squared term dropped to marginal and (ii) the Sex * language squared term became significant. In the prosocial model, the sex term dropped to non-significance. No changes in significance were observed in conduct and emotional problem models.

Table 2. Sensitivity analysis of regression models utilising observed variables with and without influential cases

|  |  | Model 2a:  Conduct Problems | Model 2b: Emotional Problems | Model 2c:  Peer Problems | Model 2d: Prosocial |
| --- | --- | --- | --- | --- | --- |
| *Multiple regression models* | | | | | |
| Unstd b, SE, p-value |  |  |  |  |  |
| Language composite |  | -0.07 (0.10)  P = .485 | -0.38 (0.17)  P = **.030** | -0.09 (0.14)  P = .511 | 0.37 (0.19)  P = .059 |
| Language composite squared |  | 0.10 (0.05)  P = .055 | 0.15 (0.08)  P = .060 | 0.18 (0.06)  P = **.005** | -0.20 (0.07)  **P = .004** |
| Reception Year SDQ |  | 0.43 (0.09)  P = **.000** | 0.11 (0.14)  P = .140 | 0.25 (0.07)  P = **.001** | -0.32 (0.06)  **P = .000** |
| SES |  | 1.36 (0.76)  P = .074 | 1.73 (1.67)  P = .302 | 2.62 (1.26)  P = **.039** | -0.65 (1.71)  P = .702 |
| Sex |  | 0.17 (0.18)  P = .352 | -0.23 (0.33)  P = .482 | 0.15 (0.23)  P = .524 | -0.71 (0.34)  **P = .038** |
| NVIQ |  | 0.00 (0.08)  P = .982 | 0.08 (0.13)  P = .554 | -0.11 (0.11)  P = .320 | 0.01 (0.14)  P = .907 |
| Sex * Language |  | 0.20 (0.17)  P = .230 | -0.13 (0.28)  P = .647 | -0.30 (0.22)  P = .180 | -0.07 (0.28)  P = .868 |
| Sex * Language sq |  | -0.10 (0.10)  P = .316 | 0.30 (0.16)  P = .061 | 0.23 (0.13)  P = .083 | 0.15 (0.14)  P = .276 |
| Sample sizes |  |  |  |  |  |
| model n; weighted n |  | 363;  6489 | 363;  6489 | 363;  6489 | 363;  6489 |
| n. Cooks d >2 |  | 28 | 2 | 6 | 2 |
| n. Standardised residuals >+/-3 |  | 8 | 3 | 5 | 2 |
| Total n. influential cases |  | 36 (9.9%) | 5 (1.3%) | 11 (3.0%) | 4 (1.1%) |
| *Multiple regression models with influential cases removed* | | | | | |
| Unstd b, SE, p-value |  |  |  |  |  |
| Language composite |  | -0.07 (0.12)  P = .546 | -0.38 (0.17)  P = **.036** | -0.12 (0.14)  P = .371 | 0.37 (0.19)  P = .056 |
| Language composite squared |  | 0.06 (0.06)  P = .284 | 0.17 (0.09)  P = .056 | 0.11 (0.06)  P = .060 | -0.18 (0.07)  P = **.014** |
| Reception Year SDQ |  | 0.42 (0.10)  P = **.000** | 0.12 (0.08)  P = .139 | 0.23 (0.07)  P = **.001** | -0.31(0.06)  P = **.000** |
| SES |  | 1.49 (0.77)  P = .055 | 1.70 (1.69)  P = .314 | 2.57 (1.26)  P = **.042** | -0.67 (1.72)  P = .696 |
| Sex |  | 0.05 (0.18)  P = .781 | -0.22 (0.34)  P = .510 | 0.10 (0.22)  P = .645 | -0.66 (0.35)  P = .059 |
| NVIQ |  | 1. (0.09)   P = .979 | 0.07 (0.13)  P = .567 | -0.07 (0.11)  P = .543 | -0.01 (0.15)  P = .960 |
| Sex * Language |  | 0.20 (0.19)  P = .279 | -0.14 (0.30)  P = .639 | -0.36 (0.22)  P = .103 | -0.02 (0.29)  P = .939 |
| Sex * Language sq |  | 0.01 (0.10)  P = .950 | 0.29 (0.18)  P = .109 | 0.28 (0.11)  P = **.014** | 0.10 (0.14)  P = .503 |
| Sample sizes |  |  |  |  |  |
| Sample n; weighted n |  | 342  ;6196 | 355  ;6313 | 355;  6327 | 358  ;6340 |
| n. Cooks d >2 |  | 29 | 1 | 9 | 1 |
| n. Standardised residuals >+/-3 |  | 8 | 3 | 5 | 1 |
| Total n. influential cases |  | 35 (10.2%) | 4 (1.1%) | 14 (3.9%) | 2 (0.6%) |

**Discussion**

None of the cases flagged as influential are due to ‘implausible’ values, as all had valid responses within the boundaries of their measures. SDQ subscale scores were within scores of 0 to 10 while standardised language composite scores were within -3.5 to 2.9 SD. Instead, these 0.6 to 10.2 % of flagged cases represent natural variation which are not well explained by the specified regression model.

The impact of flagged cases was found to be minimal. The significance of language and language interaction terms did not change when flagged cases were removed across SDQ outcomes of conduct, emotional and prosociality. The language x language term was no longer significant when flagged cases were removed in peer problems models. This indicates that the non-linear relationship for observed (as opposed to latent) peer problems is dependent on influential cases, though the significance of these terms remained marginal (p=.064; .060).

The presence of these flagged cases is consistent with limitations which we discuss in the main manuscript. Language, NVIQ, sex and SES are a limited subset of all predictors of SEB, which are known to be predicted by multiple biological, environmental and psychosocial factors. It is expectable that for some children, these factors may have nothing to do with their poor SEB, flagged as high residuals, which are datapoints very far from that predicted by current regression models. Regression models of observed (as opposed to latent) peer and conduct problems continued to exhibit influential cases, and thus may be less likely to replicate. Flagged as having a Cooks d >2, these influential cases appear in areas of the curve where datapoints are sparse, such as at the ends of the language continuum. This limitation is present despite utilising the largest and most representative cohort of children with multiple language measures to date (weighted n = 6451).

Finally, regression models utilised here assume zero measurement error, which in reality can arise from inadvertent errors from participant fatigue, inattention. questionnaire wording. They also assume that SDQ composites have the same ability to measure the construct of SEB, even as children in this cohort undergo developmental changes from Reception to Year 3. These multiple sources of measurement error attenuate regression beta estimates (Muthen, Muthen & Asparouhov, 2016; Gunole et al., 2014), from which residuals and Cooks Ds are based on. In this way, these observed variable regression results are less valid and less reliable then latent variable results of the relationship between language and SEB.

References

Guenole, N., & Brown, A. (2014). The consequences of ignoring measurement invariance for path coefficients in structural equation models. Frontiers in psychology, 5(980). doi:10.3389/fpsyg.2014.00980

Li, J., & Valliant, R. (2015). Linear regression diagnostics in cluster samples. *Journal of Official Statistics*, *31*(1), 61-75.

Muthén, B. O., Muthén, L. K., & Asparouhov, T. (2017). *Regression and mediation analysis using Mplus*. Los Angeles, CA: Muthén & Muthén.

Valliant, R., & Valliant, M. R. (2018). Package ‘svydiags’.
